# DNA methyltransferase 3a mediates developmental thermal plasticity

**DOI:** 10.1186/s12915-020-00942-w

**Published:** 2021-01-21

**Authors:** Isabella Loughland, Alexander Little, Frank Seebacher

**Affiliations:** 1 School of Life and Environmental Sciences A08, University of Sydney, Sydney, NSW 2006 Australia; 2 Department of Biology, Biosciences Complex, Queen’s University, Kingston, Ontario K7L 3N6 Canada

**Keywords:** DNA methylation, Acclimation, Transgenerational plasticity, Epigenetics, Cost of plasticity, Metabolism, Locomotor performance, Zebrafish

## Abstract

**Background:**

Thermal plasticity is pivotal for evolution in changing climates and in mediating resilience to its potentially negative effects. The efficacy to respond to environmental change depends on underlying mechanisms. DNA methylation induced by DNA methyltransferase 3 enzymes in the germline or during early embryonic development may be correlated with responses to environmental change. This developmental plasticity can interact with reversible acclimation within adult organisms, which would increase the speed of response and could alleviate potential mismatches between parental or early embryonic environments and those experienced at later life stages. Our aim was to determine whether there is a causative relationship between DNMT3 enzyme and developmental thermal plasticity and whether either or both interact with short-term acclimation to alter fitness and thermal responses in zebrafish (*Danio rerio*).

**Results:**

We developed a novel DNMT3a knock-out model to show that sequential knock-out of DNA methyltransferase 3a isoforms (DNMT3aa^−/−^ and DNMT3aa^−/−^ab^−/−^) additively decreased survival and increased deformities when cold developmental temperatures in zebrafish offspring mismatched warm temperatures experienced by parents. Interestingly, short-term cold acclimation of parents before breeding rescued DNMT3a knock-out offspring by restoring survival at cold temperatures. DNMT3a knock-out genotype interacted with developmental temperatures to modify thermal performance curves in offspring, where at least one DNMT3a isoform was necessary to buffer locomotion from increasing temperatures. The thermal sensitivity of citrate synthase activity, an indicator of mitochondrial density, was less severely affected by DNMT3a knock-out, but there was nonetheless a significant interaction between genotype and developmental temperatures.

**Conclusions:**

Our results show that DNMT3a regulates developmental thermal plasticity and that the phenotypic effects of different DNMT3a isoforms are additive. However, DNMT3a interacts with other mechanisms, such as histone (de)acetylation, induced during short-term acclimation to buffer phenotypes from environmental change. Interactions between these mechanisms make phenotypic compensation for climate change more efficient and make it less likely that thermal plasticity incurs a cost resulting from environmental mismatches.

**Supplementary Information:**

The online version contains supplementary material available at 10.1186/s12915-020-00942-w.

## Background

The capacity to remodel the thermal sensitivity of physiological rates in response to temperature cues during development can be highly advantageous because it can match phenotypes to future thermal conditions [1]. Such developmental plasticity typically results in phenotypic changes that can be relatively stable during the lifetime [2]. A potential cost is incurred when developmental conditions do not match those prevalent later in life [1]. In contrast, thermal acclimation reversibly alters the thermal sensitivity of reaction rates in response to environmental changes lasting days to weeks [3]. It is therefore possible that thermal acclimation can negate the costs of developmental plasticity [4]. The potential interaction between developmental plasticity and acclimation is important for theories explaining the evolution of plasticity, and it could increase the efficacy of plastic responses to reduce vulnerability to climate change.

Developmental plasticity can be mediated epigenetically via DNA methylation in response to internal or external environmental cues [5]. DNA methylation by DNA methyltransferases (DNMTs) can prevent binding of transcription factors to DNA and thereby alter gene expression and phenotypes [6]. There are two functionally distinct DNMTs in vertebrates: DNMT1, which is primarily associated with maintaining existing methylation marks in replicating cells, and DNMT3, which is responsible for de novo methylation in response to environmental signals [7, 8]. In stickleback (*Gasterosteus aculeatus*), for example, shifts in embryonic temperatures are correlated with different DNA methylation profiles [9]. Here, our aim was to determine whether there is a causative relationship between DNMT3 and developmental thermal plasticity and whether either or both interact with short-term acclimation to alter thermal sensitivity of physiological rates in zebrafish. Zebrafish are an ideal model organism to address these aims because they show both thermal developmental plasticity and acclimation [10], and DNMT3 gene sequences and expression profiles have been investigated [11] .

**Fig. 1:**
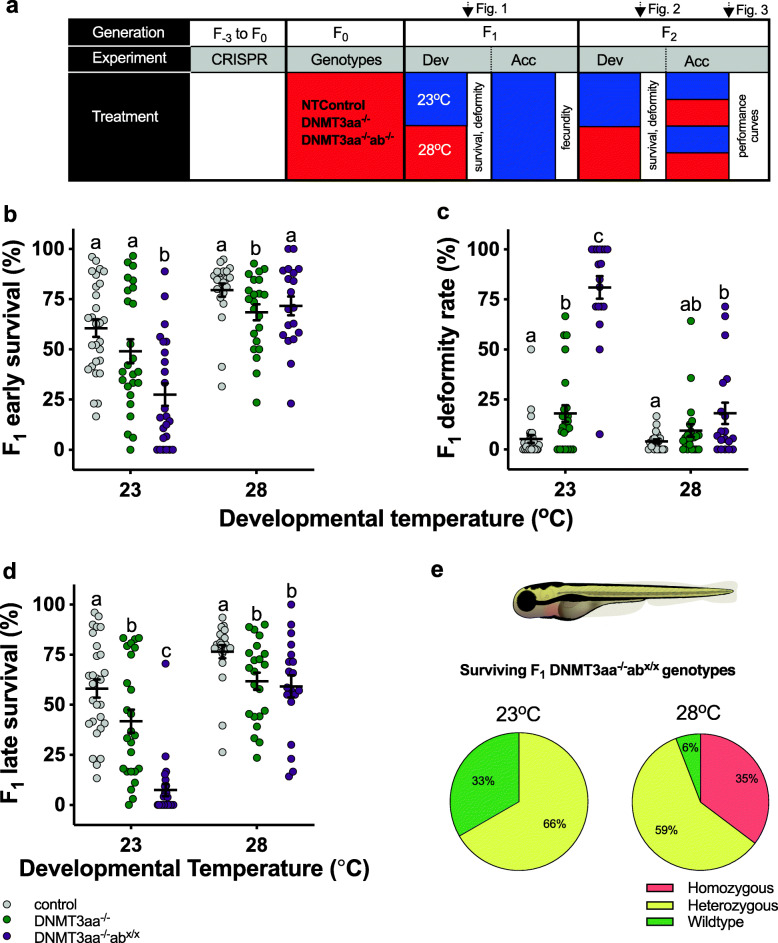
DNMT3a mediates developmental plasticity. We used CRISPR/Cas9 to produce single (DNMT3aa^−/−^) and double (DNMT3aa^−/−^ab^−/−^) knock-out lines plus a no-template control (control) (**a**). In each genotype, we tested the effect of developmental and acclimation temperatures (23 °C blue, 28 °C red) on fecundity, survival, and deformities in F_1_ and F_2_ fish. We used adult F_2_ fish to determine locomotor and metabolic thermal performance curves. The points in the experiment where data in the different figures were collected are marked on top of the panel. At 23 °C developmental temperature (**b**), survival to the 26-somite stage (early survival) was lowest in the DNMT3aa^−/−^ab^x/x^ (ab mixed genotype) fish (purple circles; *p* < 0.0001 compared to control), and survival was somewhat reduced in the DNMT3aa^−/−^ line compared to control at 28 °C (*p* < 0.04). Deformities (**c**) increased from control (grey circles) to DNMT3aa^−/−^ (green circles; *p* = 0.004) and DNMT3aa^−/−^ab^x/x^ fish (*p* < 0.0001) at 23 °C developmental temperature and were elevated in DNMT3aa^−/−^ab^x/x^ fish at 28 °C compared to control (*p* = 0.002). Survival to swim bladder inflation (late survival) (**d**) at 23 °C was lowest in DNMT3aa^−/−^ab^x/x^ fish (*p* < 0.0001 compared to control) and intermediate in DNMT3aa^−/−^ fish (*p* = 0.02 compared to control). Late survival was reduced in the two knock-out lines at 28 °C (both *p* < 0.01 compared to control). Means ± s.e. are shown in B-D (black horizontal and vertical lines, respectively), and each symbol represents data from one clutch (*n* = 19–28 clutches per treatment). Letters indicate significant differences between groups. There were no homozygous knock-out survivors (**e**) at the ab locus in the F_1_ DNMT3aa^−/−^ab^x/x^ line at 23 °C developmental temperature, but 35% of survivors were DNMT3aa^−/−^ab^−/−^ homozygous fish at 28 °C

Zebrafish have six DNMT3 genes, two paralogues of mammalian DNMT3a (DNMT3aa and DNMT3ab) and four paralogues of DNMT3b [12]. The two isoforms of DNMT3a (aa and ab) are expressed in a temperature-sensitive manner later in development (mostly > 72 h post-fertilisation) compared to DNMT3b isoforms (blastula) and are more highly expressed in developing muscle and adult fish [11, 12]. We therefore used CRISPR-Cas9 to knock-out DNMT3aa (also known as DNMT3a2 or DNMT8) and DNMT3ab (also known as DNMT3a1 or DNMT6) to test their effects on developmental plasticity. We created a single isoform DNMT3aa^−/−^ knock-out line and then generated a double isoform DNMT3aa^−/−^ab^−/−^ knockout line to determine both the individual and additive effects of DNMT3a isoforms on thermal plasticity (Fig. 1a; Additional file 1: Fig. S1).

We tested the following hypotheses: (i) DNMT3a knock-out abolishes plastic responses to different developmental temperatures (23 and 28 °C) and therefore reduces offspring fitness at novel developmental temperatures. Further, we predicted that (ii) the effects would be greater in double knock-out DNMT3aa^−/−^ab^−/−^ fish compared to the single knockout DNMT3aa^−/−^ line. If there is a reduction in developmental plasticity, we predicted (iii) that short-term acclimation can alleviate the negative effects of mismatched parent-offspring environments. Developmental plasticity and acclimation can result in a shift of thermal performance curves [4], so we tested that (iv) DNMT3a knock-out will alter the thermal sensitivity of performance-related traits following thermal acclimation of fish developed at different temperatures.

## Results

### DNMT3a mediates developmental plasticity

In offspring (F_1_) from parents raised at 28 °C (F_0_) (Fig. 1a, Additional file 1: Fig. S1), there were significant interactive effects between developmental temperature and DNMT3a genotype (Table 1). Note that at the F_1_ stage, the double knockout line comprised fish that were homozygous knockout, heterozygous knockout, and wildtype at the ab locus (ab^−/−^, ab^−/+^, and ab^+/+^, respectively, Fig. 1 and Methods). Hereafter, we use the term DNMT3aa^−/−^ab^x/x^ to denote these F_1_ fish. Our intention was to collect homozygous knock-out fish from each developmental temperature, but to our surprise, no homozygous knock-out fish survived at 23 °C (see below).

**Table 1:** Results from the statistical analysis of embryo survival and deformity. Results from permutational analyses of the effect of developmental temperature (DevT) and DNMT3a genotype (Gen) on survival rate to 26 somite stage (early survival), larval deformity rates (deformity), and survival to swim bladder inflation (late survival) in the F_1_ and F_2_ generations, as well as the effect of DNMT3a genotype on F_1_ fecundity

	Early survival		Deformity		Late survival		Fecundity
	F_1_	F_2_	F_1_	F_2_	F_1_	F_2_	F_1_
Gen	0.0074	0.54	< 0.0001	0.027	< 0.0001	0.38	0.85
DevT	< 0.0001	0.90	< 0.0001	0.94	< 0.0001	0.96	
Gen*DevT	0.034	0.85	< 0.0001	1	0.0048	0.57	

At 23 °C developmental temperatures, the F_1_ DNMT3aa^−/−^ab^x/x^ genotypes had significantly lower survival rates to the 26-somite stage (“early survival”) (*p* = 0.034; Fig. 1b; Table 1) compared to control fish and had higher deformity rates (*p* < 0.0001; Fig. 1c; Additional file 1: Fig. S2; Table 1),and lower survival rates to swim bladder inflation (6–9 days post-fertilisation, “late survival”) (*p* = 0.0048; Fig. 1d; Table 1) than DNMT3aa^−/−^ and control F_1_ fish. DNMT3aa^−/−^ genotypes also had significantly higher deformity rates and significantly lower late survival rates than control fish at 23 °C developmental temperatures, indicating an additive effect of DNMT3a isoforms (Fig. 1c, d). At 28 °C developmental temperatures, DNMT3aa^−/−^ and DNMT3aa^−/−^ab^x/x^ genotypes had significantly lower early (DNMT3aa^−/−^ only) and late survival rates than control fish, but the magnitude of the decrease was small (genotype x developmental temperature interaction; Table 1; Fig. 1b, d). DNMT3aa^−/−^ab^x/x^ fish also showed significantly higher deformity rates than control at 28 °C, but these did not significantly differ from DNMT3aa^−/−^ rates (Fig. 1c; Table 1).

The double knock-out in this experiment contained F_1_ fish with knock-out and wild-type alleles (see the “Methods” section). However, sequencing showed that in contrast to fish developed at 28 °C, there were no homozygous knock-out fish (i.e., fish with no wild-type allele present) among the surviving F_1_ that developed at 23 °C (Fig. 1e), indicating that the absence of both DNMT3a isoforms prevented development at the cooler temperature. The proportion of surviving homozygous knock-out (DNMT3aa^−/−^ab^−/−^) F_1_ offspring at 28 °C was 35% (Fig. 1e). The survival of the heterozygous and wild-type (at the ab locus) fish at 23 °C and the survival of the DNMT3aa^−/−^ab^−/−^ fish at 28 °C also indicate that the injection per se did not cause the high mortality of the DNMT3aa^−/−^ab^−/−^ fish at 23 °C; our preliminary trials also show that injection per se did not alter survival (see the “Methods” section).

### Parental acclimation rescues offspring survival

We hypothesised that acclimation of 28 °C-raised parents to 23 °C before breeding will rescue the reduced survival rates of homozygous DNMT3aa^−/−^ and DNMT3aa^−/−^ab^−/−^ offspring when developmental temperatures matched parental acclimation temperatures (23 °C).

**Fig. 2:**
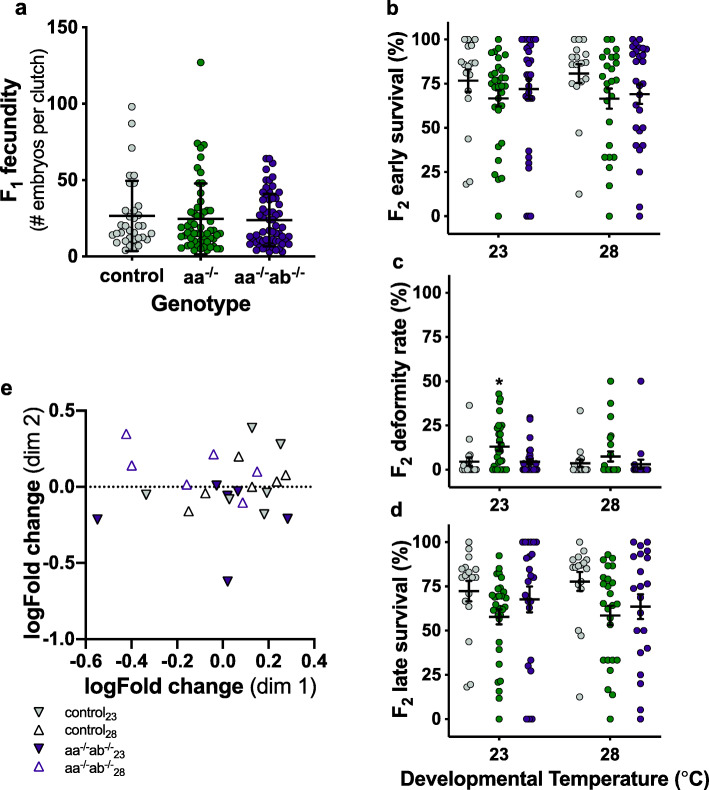
Parental acclimation rescues offspring survival. Fecundity of F_1_ fish (**a**) reared at 28 °C and acclimated to 23 °C did not differ significantly between genotypes (control = grey circles, DNMT3aa^−/−^ green circles, and DNMT3aa^−/−^ab^−/−^ purple circles). Genotype or developmental temperature had no effect on F_2_ early survival rates (**b**). There was a small but significant increase in deformity rates in DNMT3aa^−/−^ larvae at 23 °C (indicated by asterisk) (**c**). There was no significant effect of genotype or developmental temperature on late survival rates (**d**). Each circle in **a**–**d** represents an individual clutch (*n* = 17–32 clutches per treatment), and means ± s.e. are shown (black horizontal and vertical lines, respectively). Multiple dimension scaling plot (**e**) of reduced representation bisulphite sequencing results showed no global differences in methylation pattern between control and DNMT3aa^−/−^ab^−/−^ fish from both developmental temperatures (triangles = 28 °C developmental temperatures, inverted triangles = 23 °C, grey triangles = control, purple triangles = DNMT3aa^−/−^ab^−/−^)

We raised homozygous F_1_ offspring (control, DNMT3aa^−/−^, DNMT3aa^−/−^ab^−/−^) from the 28 °C developmental temperature treatment above. At 2 months of age, we acclimated these F_1_ fish to 23 °C for at least 1 month, and then bred them to produce the F_2_ generation (Fig. 1a). We found no effect of DNMT3a genotype on F_1_ fecundity (*p* = 0.85; Fig. 2a, Table 1). Surprisingly, and in contrast to the results above, when we raised offspring F_2_ fish at 23 °C and 28 °C developmental temperatures, there were no effects of developmental temperature, and neither the DNMT3aa^−/−^ nor the DNMT3aa^−/−^ab^−/−^ genotypes differed from control fish or from each other in their early (main effects and interaction *p* > 0.5; Fig. 2b, Table 1) or late survival (*p* > 0.3; Fig. 2d, Table 1). DNMT3aa^−/−^ F_2_ fish had a somewhat higher deformity rate at 23 °C developmental temperature (*p* = 0.03; Fig. 2c, Table 1).

We tested whether there were differences in whole genome DNA methylation patterns—possibly mediated by other DNMT3 enzymes—by reduced representation bisulphite sequencing of F_2_ fish. On average, 2% of regions (from a total of 4165) were differentially methylated at *p* < 0.05 between control and DNMT3aa^−/−^ab^−/−^ genotypes from different developmental temperatures (Additional file 2), but after adjusting for multiple comparisons, there were no differences in global methylation patterns between different genotypes or developmental temperatures (Fig. 2e).

### DNMT3a modulates thermal performance curves

Finally, we hypothesised that developmental temperature interacts with acclimation to modify the thermal sensitivity of physiological traits and that this interaction depends on DNMT3a. We acclimated adult F_2_ fish (i.e. three genotypes each raised at 23 and 28 °C developmental temperatures) for 3 weeks to 23 and 28 °C (i.e. 12 treatment groups total), and then tested sustained swimming performance (U_crit_) and citrate synthase activity in skeletal muscle across a range of acute test temperatures to determine performance curves [13]. We chose swimming performance as a response measure because it is an integrated physiological trait that is closely related to fitness [14] and citrate synthase activity because it is an indicator of mitochondrial density and hence aerobic metabolic capacity [15].

**Fig. 3:**
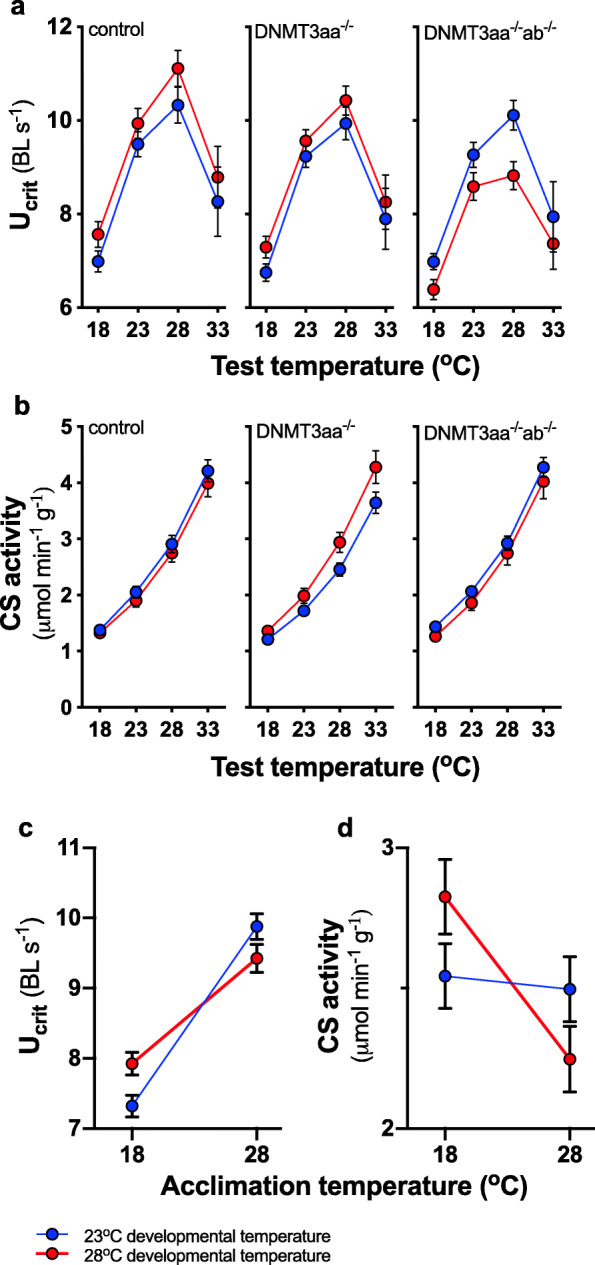
DNMT3a modulates thermal performance curves. A significant interaction between genotype and developmental temperature manifests as a progressive decline in swimming performance (U_crit_) (**a**) of warm-developed fish (red symbols and lines) from control to DNMT3aa^−/−^ and to DNMT3aa^−/−^ab^−/−^ where U_crit_ was lowest. The genotype x developmental temperature interaction determining citrate synthase (CS) activity (**b**) results from higher activity in warm-developed DNMT3aa^−/−^ fish compared to cold-developed fish. As for U_crit_, activity of warm-developed DNMT3aa^−/−^ab^−/−^ fish was lowest but similar to control. Marginal means (± s.e.) are shown across both acclimation treatments in **a** and **b**. Sample sizes were *n* = 12 for U_crit_ (24 for marginal means shown) and *n* = 8–9 for CS activity (16–18 for marginal means). Developmental temperature interacted with acclimation temperature to determine U_crit_ (**c**), and CS activity (**d**); marginal means (± s.e.) across all genotypes and test temperatures are shown

The interaction between genotype and developmental temperature significantly modified U_crit_ (*p* = 0.01; Fig. 3a, Table 2). U_crit_ in fish from the 28 °C developmental temperature was highest in control fish, but it decreased in DNMT3aa^−/−^ fish and was lowest DNMT3aa^−/−^ab^−/−^ fish; the DNMT3aa^−/−^ab^−/−^ genotype was the only one where U_crit_ in 23 °C-developed fish exceeded that of 28 °C-developed fish across all acute test temperatures (Fig. 3a). Hence, at least one isoform of DNMT3a was necessary to maintain performance as developmental temperatures increased and mismatched the parental acclimation temperature of 23 °C. There were no significant interactions between genotype and acclimation temperature (Table 2).

**Table 2:** Results from the statistical analysis of metabolic and locomotor performance. Results from the permutational analysis of the effect of DNMT3a genotype (Gen), developmental temperature (DevT), adult acclimation temperature (AccT), and acute test temperature (TestT) on sustained swimming performance (U_crit_) and citrate synthase activity (CS)

	U_crit_	CS
Gen	0.02	1
DevT	0.5	0.7
AccT	< 0.0001	0.0008
TestT	< 0.0001	< 0.0001
Gen*DevT	0.01	0.02
Gen*AccT	0.4	0.9
DevT*AccT	< 0.0001	0.01
Gen*TestT	0.08	1
Acc*TestT	< 0.0001	0.2
Gen*DevT*AccT	0.3	0.9
Gen*DevT*TestT	0.3	0.1
Gen*AccT*TestT	0.3	0.4

Citrate synthase activity was also modified significantly by the interaction between DNMT3a genotype and developmental temperature (*p* = 0.02; Fig. 3b, Table 2), and activity in warm-developed DNMT3aa^−/−^ fish was higher than in cold-developed fish. As for U_crit_, activity of warm-developed DNMT3aa^−/−^ab^−/−^ fish was lowest, but similar to control.

The interaction between developmental and acclimation temperatures significantly modified U_crit_ (*p* < 0.0001; Table 2) and citrate synthase activity (*p* = 0.01; Table 2), increasing performance when the two temperatures were mismatched (Fig. 3c, d). Both U_crit_ and citrate synthase activity changed with test temperature (Table 2). The full data set showing individual values for U_crit_ and citrate synthase activity is given in Additional file 1: Fig. S3 and Additional file 1: Fig. S4.

## Discussion

We have shown that DNMT3a mediates the capacity for developmental plasticity, with effects extending from embryonic survival and the integrity of developmental programs to whole animal physiological performance in adulthood. There was an additive effect of DNMT3aa and DNMT3ab isoforms suggesting that they have similar functions and that at least one isoform is necessary for survival at novel temperatures during development. Interestingly, acclimation of parents rescued the capacity to respond to novel developmental temperatures in DNMT3a knockout embryos. These data show that short-term acclimation of parents alleviated the negative effects of single (DNMT3aa^−/−^) and double (DNMT3aa^−/−^ab^−/−^) knock-out on development at novel, cool temperatures in the subsequent generation.

The mechanism(s) that mediate this “rescue” effect of acclimation are not clear. Our RRBS data suggest that different genotypes and developmental temperatures were not associated with differences in genome-wide methylation patterns. The reason for this result may be that reduced representation bisulphite sequencing and the short (100 bp) probes it uses to interrogate genomic methylation patterns did not have sufficient resolution to detect differentially methylated regions with statistical confidence [16]. Our data indicate that there were differentially methylated individual loci, and it may be worth to follow these up with a more targeted approach. Additionally, proteomics analyses of differential protein levels could identify candidates.

Acclimation and DNMT3a genotype both modified the effect of developmental temperature on embryonic survival and adult phenotypes, but acclimation occurred regardless of DNMT3a genotype indicating that it is mediated by different underlying mechanisms. Potential mechanisms conferring the rescuing effect of parental acclimation on offspring include differential acetylation of proteins. Histone (de)acetylation is important for mediating thermal acclimation in zebrafish [17], and acetylation marks can be passed between generations [18]. Furthermore, DNA methylation and histone modifications (acetylation and methylation) can interact to determine phenotypic responses [19]. However, neither DNMT3a genotype nor developmental temperature interacted with test temperature, indicating that neither modified the acute thermal sensitivity of swimming performance and citrate synthase activity. It is interesting to note that acclimation is the only treatment that altered responses to acute temperature changes. These results indicate that DNA methylation is relatively less important than alternative epigenetic mechanisms in modifying acute thermal sensitivity.

Discovering the mechanisms that mediate plasticity is important for understanding its evolution, because phenotypic responses can be linked to particular proteins. The evolutionary history of regulators such as DNMT and histone modifiers are well resolved [20, 21] and can thereby shed light on the evolution of phenotypic plasticity. Linking plasticity to regulators such as DNMT3 also defines the temporal dynamics with which phenotypes can change. For example, DNA methylation occurs at particular stages during development [6], so there is a strong intergenerational and developmental signal in the actions of DNMT3. However, our finding that short-term acclimation of parents can modulate the effects of DNMT3 and affect subsequent generations means that phenotypes are determined by interacting mechanisms, so mismatched parent-offspring environmental conditions are not necessarily detrimental. These results indicate that developmental and transgenerational plasticity is less susceptible to carry a cost. Environmental mismatches are viewed as a principal cost of developmental and transgenerational plasticity [1, 22], but if mismatched phenotypes are rescued by short-term acclimation, the evolutionary and ecological importance of that cost is much reduced.

## Conclusions

Our data show that responses to changing environments occur at different “superimposed” temporal scales, much like a Fourier series in physics. Longer-term environmental signals elicit transgenerational and developmental responses, which are “corrected” by short-term acclimation, thereby reducing potential costs of plasticity resulting from environmental mismatches. Developmental plasticity is mediated by DNA methylation via DNMT3a activity. An important next step to understand the temporal dynamics of plastic responses would be to confirm the mechanisms (e.g. histone acetylation) promoting the “rescue” of offspring from mismatched parent-offspring environments. Determining these mechanistic relationships will be invaluable to understand the time course of plastic responses relative to the rate of environmental change, including climate change.

## Methods

### Animals and husbandry

Wild-type (WT) TAB strain adult zebrafish of mixed sex were obtained from the Garvan Institute of Medical Research (Sydney, Australia) and used as parents to produce experimental fish. Adult fish were housed in clear plastic tanks (350 × 200 × 260 mm) within a custom-built zebrafish recirculating system [23]. Tanks were maintained at 28.5 °C unless otherwise specified, which was monitored using temperature data loggers (Hobo MX2201, OneTemp, Australia). Adult fish were fed with fish flakes (TetraMin Tropical Flakes, Tetra, USA) once per day and with newly hatched *Artemia* (Ocean Nutrition, USA) 5–6 times per week. Fish were exposed to a 14:10 h light to dark photoperiod and kept at densities of 15–25 fish per tank.

### Generating DNMT3a knock-out lines with CRISPR/Cas9

Single guide RNAs (sgRNAs) were designed using the Benchling CRISPR gRNA Design tool (http://www.benchling.com). The tool scans nucleotide sequences to detect base pair sites within a desired region that precede a NGG protospacer-adjacent motif (PAM). Potentially suitable sites are ranked based on on-target cleavage efficiency and potential off-target effects. We selected three sites within exon 6 of DNMT3aa (Ensembl DNMT3aa-201; also known as DNMT3a2 or DNMT8) and three sites within exon 7 or 8 of DNMT3ab (Ensembl DNMT3ab-002; also known as DNMT3a1 or DNMT6) based on the best on-target and off-target scores (Additional file 1: Table S1). We selected exons that occur early in the coding sequence to increase disruption by indels of the amino acid sequences in the resulting peptide. We designed primers to target each site and synthesised sgRNAs using the Invitrogen GeneArt Precision gRNA Synthesis kit (ThermoFisher Scientific, USA). We also synthesised a no-template control (control) sgRNA (TACCTCAGTTACAATTTATA) that has no high fidelity targets within the zebrafish genome [24]. The quality of sgRNAs was verified using a Bioanalyser 2100 (Agilent, USA), and concentration was measured using a Invitrogen Qubit (ThermoFisher Scientific, USA), following the manufacturers’ instructions.

sgRNA (200–500 ng/μl) and Cas9 protein (1 mM, EnGen, New England Biolabs, UK) were mixed at a 1:1 ratio to form a stable ribonucleoprotein (RNP) with 0.1% phenol red and stored at − 80 °C for a maximum of 3 weeks before injection. Breeding tanks (1.7 l, Techniplast, Italy) were set up the night before injection and contained a 1:1 or 1:2 sex ratio of males to females, which were separated by a clear divider. Dividers were removed at first light the following day, and adults were left undisturbed to breed for ~ 20 min. Fertilised eggs were collected immediately upon laying and ~ 1 ng of RNP was injected (using a Pneumatic PicoPump PV820, World Precision Instruments, USA) into one-cell stage embryos. Embryos were plated in E3 medium (5 mM NaCl, 0.17 mM KCl, 0.33 mM CaCl_2_, 0.33 mM MgSO_4_, 0.0003% methylene blue, 0.01% penicillin-streptomycin in milli-Q water, pH 7.4 with sodium bicarbonate) and incubated at 28.5 °C (0.1 °C accuracy, AE-PI-100 portable incubator, A&E Lab instruments, China). Twenty-four-hour post-fertilisation (hpf) survival rates were recorded for each sgRNA. Additionally, we assessed the possible impact of microinjection on survival of larvae by conduction three trials in which we reared eggs/larvae without (total 168 eggs) and with microinjection (total 142 eggs) in parallel. The resulting survival rates after 24 h were 78.7% (± 5.7 [s.e.]) for non-injected eggs and 75.5% (± 7.2 [s.e.]) for injected eggs indicating that injection per se did not alter survival.

DNA cleavage efficiency was determined using the T7 endonuclease I (T7E1) assay following published protocols [25]. T7E1 (New England Biolabs, USA) cleaves imperfectly matched DNA strands so that the fraction cleaved is proportional to the efficiency of gene editing. DNA was extracted from 18 to 21 7-day-old larvae from each sgRNA treatment (using the Tissue Extract-N-Amp DNA Extraction kit XNAT2, Sigma Aldrich, Australia). DNA was purified (Monarch PCR & DNA Cleanup kit, New England Biolabs, USA) and concentration and quality were determined using a Nanodrop spectrophotometer (Thermofisher Scientific, USA). Twenty-five microliters of PCR reactions were set up with Q5 Hot Start High-Fidelity Mast Mix (M0494, New England Biolabs, USA), ~ 100 ng of genomic DNA and 10 μM forward and reverse primers for each sgRNA (see Additional file 1: Table S1 for primer sequences), and PCR reactions were run (in a Heal Force K960 thermocycler, China) according to published cycling conditions [25]. PCR products were purified, and ~ 400 ng was annealed in the thermocycler, digested with T7E1, and DNA fragments were analysed in a Fragment Analyser (Agilent, USA). PCR products from control (no template)-injected larvae were included as a negative control, and PCR products from each sgRNA that were not digested with T7E1 were included as experimental controls. The relative concentrations of breakdown products allowed us to determine the efficiency of gene modification between sgRNA candidates. We selected the sgRNAs for each isoform that had the greatest mutagenesis efficiency and embryo survival.

Larvae were reared following published protocols [26]. Larvae were fed powdered spirulina algae (Sera, Germany) from 5 to 21 days post-fertilisation (dpf), filtered paramecia from 5 to 8 dpf twice per day, and 24-h-old *Artemia* larvae (Ocean Nutrition, California, USA) from 9 dpf. We genotyped 2–3-month-old fish at each generation according published protocols [27]. Cas9 activity continued beyond the first cell division so that the indels produced mosaic knock-out fish with more than the two different knock-out sequences [28]. We therefore used CRISP-ID software [29] to screen for homozygous knock-out fish (two or more alleles containing indels but no wild-type [WT] sequence present) or heterozygous (one or more alleles containing indels but WT allele still present) F_0_ fish. We bred the DNMT3aa homozygous and heterozygous fish and genotyped to select homozygous fish (DNMT3aa^−/−^; see Additional file 1: Fig. S5 for example sequences, and Additional file 3 for representative examples of sequences for all genotypes). We screened each control fish for mutations in both DNMT3aa and DNMT3ab to ensure that they were WT in both regions. DNA was extracted from fin clips (using the Tissue Extract-N-Amp DNA Extraction kit XNAT2, Sigma Aldrich, Australia), and all Sanger sequencing was conducted by the Australian Genome Research Facility (AGRF; Melbourne, Australia).

### Experiment 1: DNMT3a mediates developmental plasticity

Our first experiment aimed to determine the interaction between DNMT3a genotype and developmental temperature on embryo survival. We bred homozygous control and DNMT3aa^−/−^ fish to obtain the F_1_ generation. We injected F_1_ DNMT3aa^−/−^ embryos with DNMT3ab sgRNA/Cas9 to create a double knock-out DNMT3aa^−/−^ab^x/x^line. The DNMT3aa^−/−^ab^x/x^ genotype was comprised of an unknown proportion of wild-type, heterozygous (i.e. one wild-type allele present), and homozygous knock-out (i.e. no wild-type allele present) fish at the ab locus. After the breeding experiment (below), we therefore genotyped the surviving DNMT3aa^−/−^ab^x/x^ larvae (*n* = 17–18) from both developmental temperatures to determine genotypes; 9 dpf larvae were euthanised with an overdose of MS-222 (0.25 g/L, pH 7.0), and whole larvae were used for DNA extraction and sequencing as above.

For the breeding experiment, we divided clutches from each genotype (control, DNMT3aa^−/−^, DNMT3aa^−/−^ab^x/x^) into two developmental temperature treatments (23.5 ± 0.5 °C and 28.5 ± 0.5 °C; we refer to these treatments as 23 and 28 °C, respectively, in the text). We raised embryos at these temperatures with clutches separated into different tanks (*n* = 19–28 clutches per treatment) and recorded embryo survival and deformity rates.

We recorded the survival rates of each clutch of embryos at the 26 somite stage [30], which was ~ 24 hpf at 28.5 °C and ~ 48 hpf at 23.5 °C (“early survival”). We also measured survival rates within each clutch at the time of swim bladder inflation (6 dpf at 28 °C and 9 dpf at 23 °C; “late survival”). Additionally, we recorded rates of deformity until 6–9 dpf as the total number of deformed larvae up to the swim bladder stage divided by the total number of embryos surviving to the 26 somite stage. We classified a larva as deformed when it had a significant and non-viable morphological deviation from the standard phenotype. Deformities were variable and were apparent at different developmental stages (Additional file 1: Fig. S2), but all forms lead to premature larval death, although not all dead larvae were deformed.

### Experiment 2: Parental acclimation rescues offspring survival

Our second experimental aim was to determine whether parental acclimation alters the effect of DNMT3a knock-out on embryo fitness at different developmental temperatures.

We reared F_1_ larvae of each genotype (control, DNMT3aa^−/−^ and DNMT3aa^−/−^ab^x/x^) at 28 °C for 2 months. We then changed the temperature gradually (1 °C/day) to 23 °C and kept fish at that temperature for at least one month. Fish were dispersed across at least 4 tanks per treatment. At 3 months of age, we genotyped DNMT3aa^−/−^ab^x/x^ F_1_ fish from fin clips, as above, and screened for homozygous knock-out individuals (DNMT3aa^−/−^ab^−/−^) to use for subsequent breeding and experiments. We then bred F_1_ fish of each genotype at 23 °C and again split egg clutches (*n* = 17–32) into 23 and 28 °C developmental temperature treatments. We recorded parental (F_1_) fecundity (total embryos produced per female) and embryo (F_2_) survival rates and deformity as described above for experiment 1.

DNA methylation patterns of F_2_ fish were determined by reduced representation bisulphite sequencing in skeletal muscle from control and DNMT3aa^−/−^ab^−/−^ adult fish raised at 23 and 28 °C developmental temperatures (*n* = 6 per treatment, 24 samples in total). DNA extraction, reduced-representation bisulphite sequencing, and bioinformatic analyses were conducted by the AGRF (Melbourne, Australia). The AGRF Ltd. is an Illumina Certified Service Provider, and it is accredited in the field of Biological Testing (Scope: DNA Analysis) by the National Association of Testing Authorities (NATA); all work was conducted according to the ISO17025: 2005 standard. Briefly, bisulphite-treated reduced representation genomic DNA libraries were produced using the Ovation RRBS Methyl-Seq System (Tecan, USA) on Mspl-digested gDNA, and sequenced using the Illumina NovaSeq platform. Samples were split between two lanes (two duplicates per sample, so 24 samples per lane). The primary sequence data were generated using the Illumina bcl2fastq 2.20.0.422 pipeline.

Raw sequences were trimmed, low-quality fragments were removed, and clean reads were mapped to the zebrafish reference genome using Bismark v0.21.0 [31]. Alignments were performed with the Bowtie2 v2.3.4 [32] aligner, and PCR duplicates were removed. An average of 16,830,254 ± 838,201 (mean ± s.d.) 100 bp single reads were obtained per sample which covered an average of 240,325 ± 9855 (mean ± s.d.) CpG sites. CpG sites were defined as having at least 10 total cytosines (both methylated and non-methylated). Differentially methylated regions were detected and quantified using the EdgeR package [33]. edgeR linear models were used to fit the total read count (methylated plus unmethylated) at each genomic locus. Differential methylation was assessed by likelihood ratio tests, and FDR values of less than 0.05 were considered to indicate significant differentially methylated regions (DMR). Data are presented as multiple dimension scaling plots.

### Experiment 3: DNMT3a modulates thermal performance curves

We tested whether there was an interaction between developmental temperature, acclimation temperature, and DNMT3a genotype on whole-animal performance. We reared F_2_ fish of each genotype (control, DNMT3aa^−/−^ and DNMT3aa^−/−^ab^−/−^) from experiment 2 at their respective developmental temperatures for ~ 3 months, and then acclimated fish from each genotype x developmental temperature combination to either 18 or 28 °C (± 0.5 °C) for 3–4 weeks (12 treatments in total). Fish were dispersed across three tanks per treatment.

Following acclimation, we measured sustained swimming performance (U_crit_; in *n* = 12 fish per treatment) and citrate synthase (CS) activity (duplicate assays in *n* = 8–9 fish per treatment) at 18, 23, 28, and 33 °C acute test temperatures. We used published protocols to determine U_crit_ [34] and CS activity [35]. Following swim trials, each fish was weighed on an electronic balance, and body length was determined from photographs (using Image J software, National Institute of Health, USA). U_crit_ is shown as standard body length s^−1^ (BL s^−1^). Each individual was measured at each acute test temperature (18, 23, 28, and 33 °C), and the order of test temperatures was randomised. To track fish between measurements, we kept individuals in cylindrical plastic containers (1 l volume) that were submerged in tanks within the recirculating system. The containers had vertical slits that allowed olfactory and visual contact between fish, but prevented egress of fish. There were at least 48 h between swimming trials.

### Statistical analysis

We analysed all data with permutational analyses of variance using the lmPerm package in R [36]. lmPerm uses type III sums of squares and implements ANOVA models but calculates probabilities based on the number of randomised data sets that show the same or greater effect as the measured data set, divided by the total number of permutations. Hence, permutational analyses use the data per se without assumptions about underlying distributions. Permutational analyses do not have any associated test-statistic and are often preferable to frequentist statistics [37], particularly when data stem from mixed or unknown distributions [38].

For the analysis of experiment 1, we tested whether there was an effect of DNMT3a genotype (control, DNMT3aa^−/−^ and DNMT3aa^−/−^ab^x/x^) and developmental temperature (23 and 28 °C) on F_1_ embryo early survival rates, deformity rates, and later survival rates. The date of fertilisation and parental breeding pair ID were included as random factors in the model.

We used a similar analysis in experiment 2 to test the hypothesis that the effects of DNMT3a genotype and developmental temperature on embryo fitness was altered by prior acclimation of parents to 23 °C. We also conducted a one-factor permutational analysis of variance to test whether there was an effect of parental (F_1_) genotype on fecundity (total embryos produced).

For experiment 3, we analysed the effects of DNMT3a genotype, developmental temperature, acclimation temperature, and acute test temperature on U_crit_ and CS activity of F_2_ fish. For analyses of U_crit_, Fulton’s condition factor [100*(mass/length^3^)] was used as a covariate to account for any variation explained by differences in body shape, and test temperature was included as a quadratic term (TestT + TestT^2^). Changes in citrate synthase activity with test temperature were approximately linear, so we included test temperature as a linear term (TestT) only. In both analyses, we used a random intercept model with Fish ID as a random factor to account for repeated measures of individuals at different test temperatures. For all analyses, we compared marginal means with post hoc permutational analyses where there were significant effects.

## Supplementary Information

**Additional file 1.** Supplementary data (Figures S1-S5; Table S1).

**Additional file 2.** Reduced representation bisulphite sequencing, differentially methylated regions data.

**Additional file 3.** Representative examples of sequences for each genotype.

## Data Availability

All data generated or analysed during this study are included in this published article [and its supplementary information files].
