# Supplementary material for: DNA methyltransferase 3a mediates developmental thermal plasticity
Source: BMC Biol. 2021 Jan 21;19:11. doi: 10.1186/s12915-020-00942-w (PMC7819298; doi:10.1186/s12915-020-00942-w)
Supplement: Supplementary file 1 — Additional file 1. Supplementary data (Figures S1-S5; Table S1). [file 12915_2020_942_MOESM1_ESM.docx]

**Supplementary Materials for**

**DNA methyltransferase 3a mediates thermal plasticity**

Isabella Loughland^1^, Alexander Little^2^ and Frank Seebacher^1*^

*^1^School of Life and Environmental Sciences A08, University of Sydney, NSW 2006, Australia*

*^2^Department of Biology, Biosciences Complex, Queen's University, Kingston, Ontario, Canada K7L 3N6*

Correspondence to: frank.seebacher@sydney.edu.au


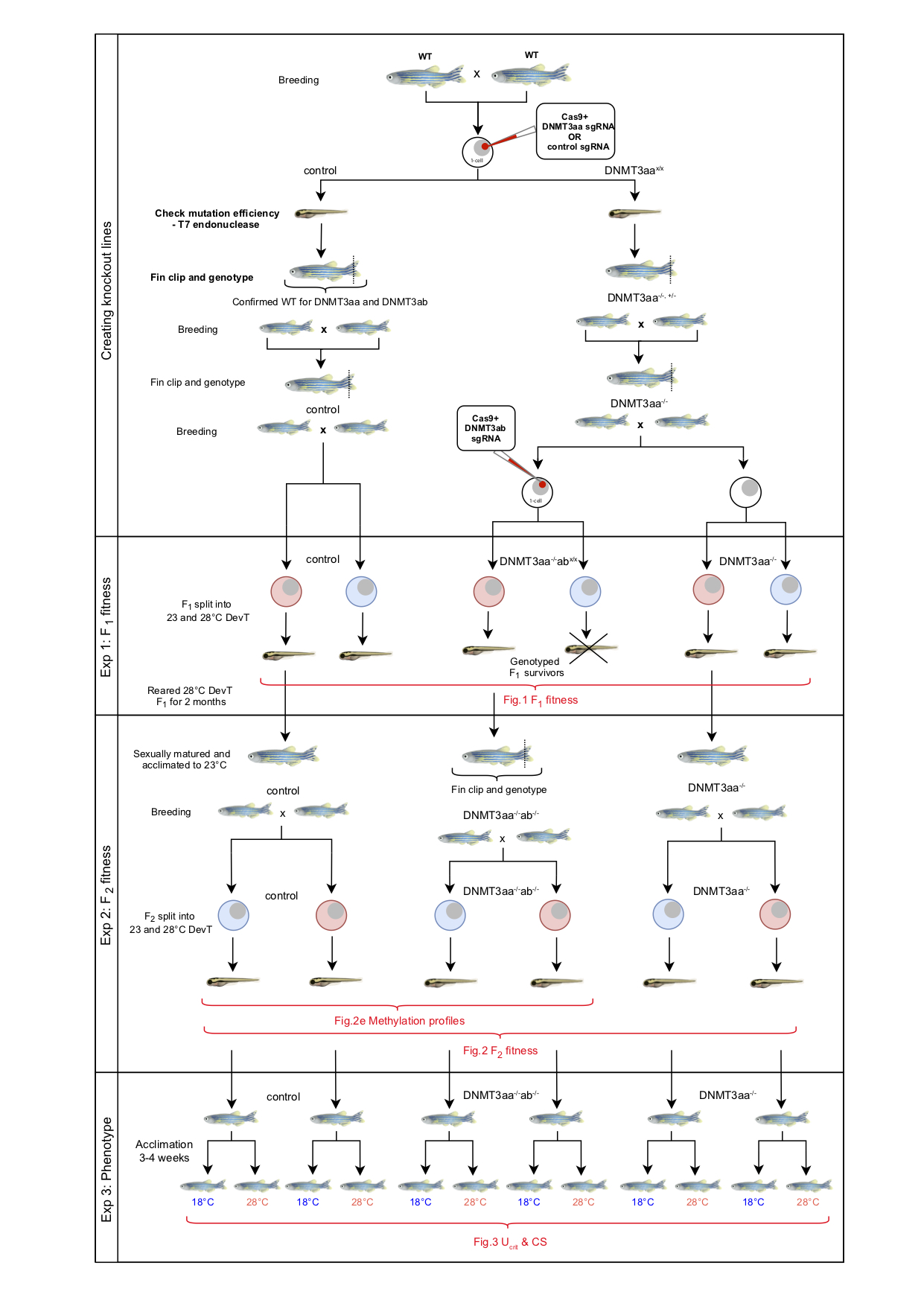


**Fig. S1 Schematic depiction of the experimental design.** Different experiments in F_1_ and F_2_ generations are shown on the left, and red writing indicates collection points of data presented in the main Figures.


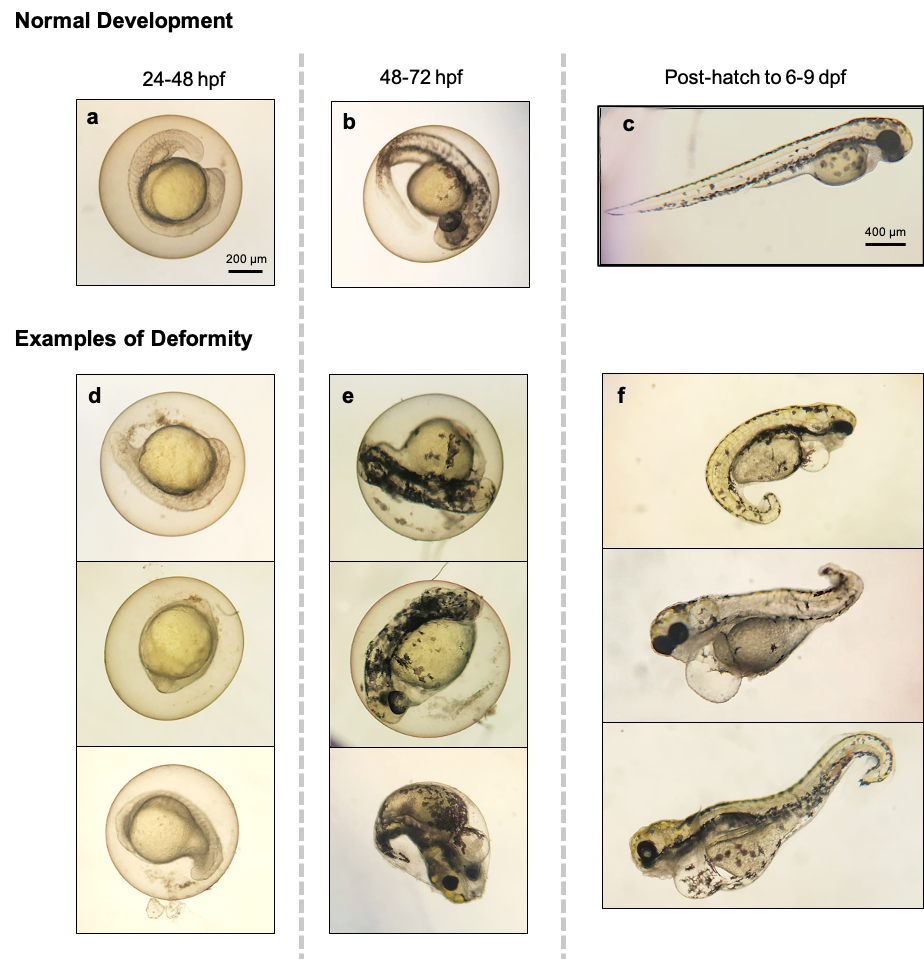


**Fig. S2 Examples of larval deformity.** Confocal images of (A-C) normal developing larvae at **a**, 24-48 hpf, **b**, 48-72 hpf, and **c**, at 6-9 dpf. Examples of deformities at **d**, 24-28 hpf, **e**, 48-72 hpf, and **f**, 6-9 dpf.

**Fig. S3** **Sustained swimming performance (U_crit_).** The effects of DNMT3a genotype and acclimation temperature (18^o^C acclimation = blue circles; 28^o^C acclimation = red circles) on fish developed at 23^o^C (**A**) and 28^o^C (**B**). Sample sizes are n=12 fish per treatment group, and data from individual fish are shown as well as means ± s.e.

**Fig. S4** **Citrate synthase (CS) activity.** The effects of DNMT3a genotype and acclimation temperature (18^o^C acclimation = blue circles; 28^o^C acclimation = red circles) on fish developed at 23^o^C (**A**) and 28^o^C (**B**). Sample sizes are n=12 fish per treatment group, and data from individual fish are shown as well as means ± s.e.

*
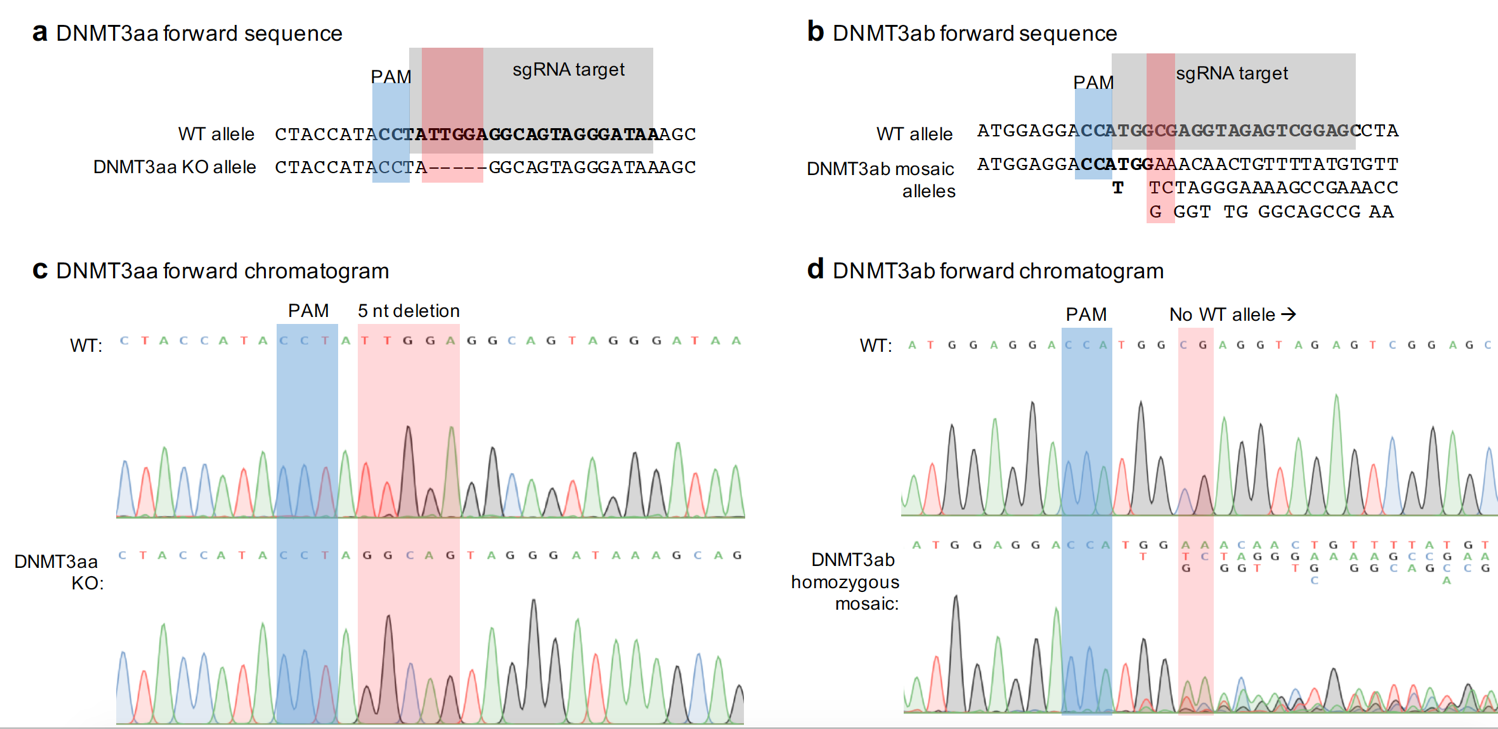
*

**Fig. S5 DNA sequences and chromatograms of DNMT3a knockout fish**. Example diagram of the single guide RNA (sgRNA) target on the aa (**A**) and ab (**B**) alleles and their respective chromatograms (**C** and **D**) showing comparisons between knock-out and wild-type sequences. Protospacer-adjacent motif recognition sites (PAM, blue shaded areas), and indel mutation sites (red shaded area) are shown.

**Table S1 sgRNA candidates targeting DNMT3a.** Benchling software (<http://www.benchling.com>) was used to find single guide RNA (sgRNA) candidates that precede a NGG protospacer-adjacent motif (PAM) in exon 8 of DNMT3aa isoform and exon 7 and 8 of DNMT3ab isoform. We tested three possible targets for each isoform with both a specificity (off-target effects) and efficiency (on-target cleavage) score of >50, and selected sgRNAs (bolded) for each isoform that had the lowest embryo toxicity and highest mutagenesis efficiency.

| sgRNA | Position | Strand | Sequence | PAM | Specificity score | Efficiency score |
| --- | --- | --- | --- | --- | --- | --- |
| **DNMT3aa_1** | **77650** | **-1** | **TTATCCCTACTGCCTCCAAT** | **AGG** | **92.1** | **90.0** |
| DNMT3aa_2 | 77562 | -1 | TGTCACTCATTTGAACAAGG | CGG | 83.7 | 65.7 |
| DNMT3aa_3 | 77649 | 1 | CTTCCTACCATACCTATTGG | AGG | 95.1 | 50.2 |
|  |  |  |  |  |  |  |
| DNMT3ab_1 | 86998 | -1 | GCTCGTTAATGCCAAAACCT | CGG | 93.6 | 70.2 |
| **DNMT3ab_2** | **84000** | **-1** | **GCTCCGACTCTACCTCGCCA** | **TGG** | **97.8** | **68.3** |
| DNMT3ab_3 | 87044 | 1 | AGTTACGAGGATACTCCTGG | TGG | 95.3 | 53.6 |
