# Supplementary material for: DNA methyltransferase 3a mediates developmental thermal plasticity
Source: BMC Biol. 2021 Jan 21;19:11. doi: 10.1186/s12915-020-00942-w (PMC7819298; doi:10.1186/s12915-020-00942-w)
Supplement: Supplementary file 3 — Additional file 3. Representative examples of sequences for each genotype. [file 12915_2020_942_MOESM3_ESM.pdf]

CLUSTAL 2.1 MULTIPLE SEQUENCE ALIGNMENT

File: /Users/isabellaloughland/Google Drive/FrankSeebacher3/AA_alignment_3.ps   Date: Mon Feb 26 15:48:34 2018

Page 1 of 15 ; aa = DNMT3aa$^{-/-}$, NC = no template control

```
              ****************  **********************************************************************************************************************************
aa12_A11     --TCTACGCTACATTTCAAGTCAATATTTACCTAAACACAAACATCAGTCCGTATTTCTGGAGAGGAGTTCAATACTGCATCTGCTTTCGATTTGCAGTGAAAATCCTGTGAGACACTACTGTGCCCTGTGTGTCTATATTTATCCTTGT   148
aa21_B08     -CTCTACGCTACATTTCAAGTCAATATTTACCTAAACACAAACATCAGTCCGTATTTCTGGAGAGGAGTTCAATACTGCATCTGCTTTCGATTTGCAGTGAAAATCCTGTGAGACACTACTGTGCCCTGTGTGTCTATATTTATCCTTGT   149
aa24_B11     -CTCTACGCTACATTTCAAGTCAATATTTACCTAAACACAAACATCAGTCCGTATTTCTGGAGAGGAGTTCAATACTGCATCTGCTTTCGATTTGCAGTGAAAATCCTGTGAGACACTACTGTGCCCTGTGTGTCTATATTTATCCTTGT   149
aa11_A10     TCTCTACGCTACATTTCAAGTCAATATTTACCTAAACACAAACATCAGTCCGTATTTCTGGAGAGGAGTTCAATACTGCATCTGCTTTCGATTTGCAGTGAAAATCCTGTGAGACACTACTGTGCCCTGTGTGTCTATATTTATCCTTGT   150
aa25_B12     --TCTACGCTACATTTCAAGTCAATATTTACCTAAACACAAACATCAGTCCGTATTTCTGGAGAGGAGTTCAATACTGCATCTGCTTTCGATTTGCAGTGAAAATCCTGTGAGACACTACTGTGCCCTGTGTGTCTATATTTATCCTTGT   148
aa13_A12     --TCTACGCTACATTTCAG-TCAATATTTACCTAAACACAAACATCAGTCCGTATTTCTGGAGAGGAGTTCAATACTGCATCTGCTTTCGATTTGCAGTGAAAATCCTGTGAGACACTACTGTGCCCTGTGTGTCTATATTTATCCTTGT   147
aa38_D01     -CTCTACGCTACATTTCAAGTCAATATTTACCTAAACACAAACATCAGTCCGTATTTCTGGAGAGGAGTTCAATACTGCATCTGCTTTCGATTTGCAGTGAAAATCCTGTGAGACACTACTGTGCCCTGTGTGTCTATATTTATCCTTGT   149
aa45_D08     --TCTACGCTACATTTCAAGTCAATATTTACCTAAACACAAACATCAGTCCGTATTTCTGGAGAGGAGTTCAATACTGCATCTGCTTTCGATTTGCAGTGAAAATCCTGTGAGACACTACTGTGCCCTGTGTGTCTATATTTATCCTTGT   148
aa48_D11     -CTCTACGCTACATTTCAAGTCAATATTTACCTAAACACAAACATCAGTCCGTATTTCTGGAGAGGAGTTCAATACTGCATCTGCTTTCGATTTGCAGTGAAAATCCTGTGAGACACTACTGTGCCCTGTGTGTCTATATTTATCCTTGT   149
aa31_C06     TCTCTACGCTACATTTCAAGTCAATATTTACCTAAACACAAACATCAGTCCGTATTTCTGGAGAGGAGTTCAATACTGCATCTGCTTTCGATTTGCAGTGAAAATCCTGTGAGACACTACTGTGCCCTGTGTGTCTATATTTATCCTTGT   150
aa49_D12     -CTCTACGCTACATTTCAAGTCAATATTTACCTAAACACAAACATCAGTCCGTATTTCTGGAGAGGAGTTCAATACTGCATCTGCTTTCGATTTGCAGTGAAAATCCTGTGAGACACTACTGTGCCCTGTGTGTCTATATTTATCCTTGT   149
aa10_A09     --TCTACGCTACATTTCAAGTCAATATTTACCTAAACACAAACATCAGTCCGTATTTCTGGAGAGGAGTTCAATACTGCATCTGCTTTCGATTTGCAGTGAAAATCCTGTGAGACACTACTGTGCCCTGTGTGTCTATATTTATCCTTGT   148
aa18_B05     -CTCTACGCTACATTTCAAGTCAATATTTACCTAAACACAAACATCAGTCCGTATTTCTGGAGAGGAGTTCAATACTGCATCTGCTTTCGATTTGCAGTGAAAATCCTGTGAGACACTACTGTGCCCTGTGTGTCTATATTTATCCTTGT   149
aa22_B09     -CTCTACGCTACATTTCAG-TCAATATTTACCTAAACACAAACATCAGTCCGTATTTCTGGAGAGGAGTTCAATACTGCATCTGCTTTCGATTTGCAGTGAAAATCCTGTGAGACACTACTGTGCCCTGTGTGTCTATATTTATCCTTGT   148
aa8_A07      --TCTACGCTACATTTCAAGTCAATATTTACCTAAACACAAACATCAGTCCGTATTTCTGGAGAGGAGTTCAATACTGCATCTGCTTTCGATTTGCAGTGAAAATCCTGTGAGACACTACTGTGCCCTGTGTGTCTATATTTATCCTTGT   148
aa17_B04     -CTCTACGCTACATTTCAAGTCAATATTTACCTAAACACAAACATCAGTCCGTATTTCTGGAGAGGAGTTCAATACTGCATCTGCTTTCGATTTGCAGTGAAAATCCTGTGAGACACTACTGTGCCCTGTGTGTCTATATTTATCCTTGT   149
aa56_E07     -CTCTACGCTACATTTCAAGTCAATATTTACCTAAACACAAACATCAGTCCGTATTTCTGGAGAGGAGTTCAATACTGCATCTGCTTTCGATTTGCAGTGAAAATCCTGTGAGACACTACTGTGCCCTGTGTGTCTATATTTATCCTTGT   149
aa54_E05     -CTCTACGCTACATTTCAAGTCAATATTTACCTAAACACAAACATCAGTCCGTATTTCTGGAGAGGAGTTCAATACTGCATCTGCTTTCGATTTGCAGTGAAAATCCTGTGAGACACTACTGTGCCCTGTGTGTCTATATTTATCCTTGT   149
aa16_B03     -CTCTACGCTACATTTCAAGTCAATATTTACCTAAACACAAACATCAGTCCGTATTTCTGGAGAGGAGTTCAATACTGCATCTGCTTTCGATTTGCAGTGAAAATCCTGTGAGACACTACTGTGCCCTGTGTGTCTATATTTATCCTTGT   149
aa53_E04     --TCTACGCTACATTTCAAGTCAATATTTACCTAAACACAAACATCAGTCCGTATTTCTGGAGAGGAGTTCAATACTGCATCTGCTTTCGATTTGCAGTGAAAATCCTGTGAGACACTACTGTGCCCTGTGTGTCTATATTTATCCTTGT   148
aa32_C07     -CTCTACGCTACATTTCAAGTCAATATTTACCTAAACACAAACATCAGTCCGTATTTCTGGAGAGGAGTTCAATACTGCATCTGCTTTCGATTTGCAGTGAAAATCCTGTGAGACACTACTGTGCCCTGTGTGTCTATATTTATCCTTGT   149
aa26_C01     -CTCTACGCTACATTTCAAGTCAATATTTACCTAAACACAAACATCAGTCCGTATTTCTGGAGAGGAGTTCAATACTGCATCTGCTTTCGATTTGCAGTGAAAATCCTGTGAGACACTACTGTGCCCTGTGTGTCTATATTTATCCTTGT   149
aa34_C09     -CTCTACGCTACATTTCAAGTCAATATTTACCTAAACACAAACATCAGTCCGTATTTCTGGAGAGGAGTTCAATACTGCATCTGCTTTCGATTTGCAGTGAAAATCCTGTGAGACACTACTGTGCCCTGTGTGTCTATATTTATCCTTGT   149
aa6_A05      --TCTACGCTACATTTCAAGTCAATATTTACCTAAACACAAACATCAGTCCGTATTTCTGGAGAGGAGTTCAATACTGCATCTGCTTTCGATTTGCAGTGAAAATCCTGTGAGACACTACTGTGCCCTGTGTGTCTATATTTATCCTTGT   148
ncaa2_A02    TCTCTACGCTACATTTCAAGTCAATATTTACCTAAACACAAACATCAGTCCGTATTTCTGGAGAGGAGTTCAATACTGCATCTGCTTTCGATTTGCAGTGAAAATCCTGTGAGACACTACTGTGCCCTGTGTGTCTATATTTATCCTTGT   150
aa55_E06     TCTCTACGCTACATTTCAAGTCAATATTTACCTAAACACAAACATCAGTCCGTATTTCTGGAGAGGAGTTCAATACTGCATCTGCTTTCGATTTGCAGTGAAAATCCTGTGAGACACTACTGTGCCCTGTGTGTCTATATTTATCCTTGT   150
aa42_D05     TCTCTACGCTACATTTCAAGTCAATATTTACCTAAACACAAACATCAGTCCGTATTTCTGGAGAGGAGTTCAATACTGCATCTGCTTTCGATTTGCAGTGAAAATCCTGTGAGACACTACTGTGCCCTGTGTGTCTATATTTATCCTTGT   150
NC_AA_4_D10  -CTCTACGCTACATTTCA-GTCAATATTTACCTAAACACAAACATCAGTCCGTATTTCTGGAGAGGAGTTCAATACTGCATCTGCTTTCGATTTGCAGTGAAAATCCTGTGAGACACTACTGTGCCCTGTGTGTCTATATTTATCCTTGT   148
aa37_C12     -CTCTACGCTACATTTCAAGTCAATATTTACCTAAACACAAACATCAGTCCGTATTTCTGGAGAGGAGTTCAATACTGCATCTGCTTTCGATTTGCAGTGAAAATCCTGTGAGACACTACTGTGCCCTGTGTGTCTATATTTATCCTTGT   149
aa19_B06     -CTCTACGCTACATTTCAAGTCAATATTTACCTAAACACAAACATCAGTCCGTATTTCTGGAGAGGAGTTCAATACTGCATCTGCTTTCGATTTGCAGTGAAAATCCTGTGAGACACTACTGTGCCCTGTGTGTCTATATTTATCCTTGT   149
aa33_C08     -CTCTACGCTACATTTCAAGTCAATATTTACCTAAACACAAACATCAGTCCGTATTTCTGGAGAGGAGTTCAATACTGCATCTGCTTTCGATTTGCAGTGAAAATCCTGTGAGACACTACTGTGCCCTGTGTGTCTATATTTATCCTTGT   149
NC_AA_1_A10  -CTCTACGCTACATTTCAAGTCAATATTTACCTAAACACAAACATCAGTCCGTATTTCTGGAGAGGAGTTCAATACTGCATCTGCTTTCGATTTGCAGTGAAAATCCTGTGAGACACTACTGTGCCCTGTGTGTCTATATTTATCCTTGT   149
aa28_C03     -CTCTACGCTACATTTCAAGTCAATATTTACCTAAACACAAACATCAGTCCGTATTTCTGGAGAGGAGTTCAATACTGCATCTGCTTTCGATTTGCAGTGAAAATCCTGTGAGACACTACTGTGCCCTGTGTGTCTATATTTATCCTTGT   149
aa29_C04     TCTCTACGCTACATTTCAAGTCAATATTTACCTAAACACAAACATCAGTCCGTATTTCTGGAGAGGAGTTCAATACTGCATCTGCTTTCGATTTGCAGTGAAAATCCTGTGAGACACTACTGTGCCCTGTGTGTCTATATTTATCCTTGT   150
aa30_C05     -CTCTACGCTACATTTCAG-TCAATATTTACCTAAACACAAACATCAGTCCGTATTTCTGGAGAGGAGTTCAATACTGCATCTGCTTTCGATTTGCAGTGAAAATCCTGTGAGACACTACTGTGCCCTGTGTGTCTATATTTATCCTTGT   148
aa43_D06     -CTCTACGCTACATTTCAAGTCAATATTTACCTAAACACAAACATCAGTCCGTATTTCTGGAGAGGAGTTCAATACTGCATCTGCTTTCGATTTGCAGTGAAAATCCTGTGAGACACTACTGTGCCCTGTGTGTCTATATTTATCCTTGT   149
NC_AA_5_E10  -CTCTACGCTACATTTCAAGTCAATATTTACCTAAACACAAACATCAGTCCGTATTTCTGGAGAGGAGTTCAATACTGCATCTGCTTTCGATTTGCAGTGAAAATCCTGTGAGACACTACTGTGCCCTGTGTGTCTATATTTATCCTTGT   149
aa57_E08     -CTCTACGCTACATTTCAAGTCAATATTTACCTAAACACAAACATCAGTCCGTATTTCTGGAGAGGAGTTCAATACTGCATCTGCTTTCGATTTGCAGTGAAAATCCTGTGAGACACTACTGTGCCCTGTGTGTCTATATTTATCCTTGT   149
NC_AA_2_B10  -CTCTACGCTACATTTCAAGTCAATATTTACCTAAACACAAACATCAGTCCGTATTTCTGGAGAGGAGTTCAATACTGCATCTGCTTTCGATTTGCAGTGAAAATCCTGTGAGACACTACTGTGCCCTGTGTGTCTATATTTATCCTTGT   149
aa40_D03     -CTCTACGCTACATTTCAAGTCAATATTTACCTAAACACAAACATCAGTCCGTATTTCTGGAGAGGAGTTCAATACTGCATCTGCTTTCGATTTGCAGTGAAAATCCTGTGAGACACTACTGTGCCCTGTGTGTCTATATTTATCCTTGT   149
aa44_D07     -CTCTACGCTACATTTCAAGTCAATATTTACCTAAACACAAACATCAGTCCGTATTTCTGGAGAGGAGTTCAATACTGCATCTGCTTTCGATTTGCAGTGAAAATCCTGTGAGACACTACTGTGCCCTGTGTGTCTATATTTATCCTTGT   149
aa47_D10     -CTCTACGCTACATTTCAAGTCAATATTTACCTAAACACAAACATCAGTCCGTATTTCTGGAGAGGAGTTCAATACTGCATCTGCTTTCGATTTGCAGTGAAAATCCTGTGAGACACTACTGTGCCCTGTGTGTCTATATTTATCCTTGT   149
aa20_B07     -CTCTACGCTACATTTCAAGTCAATATTTACCTAAACACAAACATCAGTCCGTATTTCTGGAGAGGAGTTCAATACTGCATCTGCTTTCGATTTGCAGTGAAAATCCTGTGAGACACTACTGTGCCCTGTGTGTCTATATTTATCCTTGT   149
aa46_D09     TCTCTACGCTACATTTCAAGTCAATATTTACCTAAACACAAACATCAGTCCGTATTTCTGGAGAGGAGTTCAATACTGCATCTGCTTTCGATTTGCAGTGAAAATCCTGTGAGACACTACTGTGCCCTGTGTGTCTATATTTATCCTTGT   150
aa14_B01     --TCTACGCTACATTTCAAGTCAATATTTACCTAAACACAAACATCAGTCCGTATTTCTGGAGAGGAGTTCAATACTGCATCTGCTTTCGATTTGCAGTGAAAATCCTGTGAGACACTACTGTGCCCTGTGTGTCTATATTTATCCTTGT   148
aa35_C10     --TCTACGCTACATTTCAAGTCAATATTTACCTAAACACAAACATCAGTCCGTATTTCTGGAGAGGAGTTCAATACTGCATCTGCTTTCGATTTGCAGTGAAAATCCTGTGAGACACTACTGTGCCCTGTGTGTCTATATTTATCCTTGT   148
aa36_C11     TCTCTACGCTACATTTCAAGTCAATATTTACCTAAACACAAACATCAGTCCGTATTTCTGGAGAGGAGTTCAATACTGCATCTGCTTTCGATTTGCAGTGAAAATCCTGTGAGACACTACTGTGCCCTGTGTGTCTATATTTATCCTTGT   150
aa58_E09     -CTCTACGCTACATTTCAAGTCAATATTTACCTAAACACAAACATCAGTCCGTATTTCTGGAGAGGAGTTCAATACTGCATCTGCTTTCGATTTGCAGTGAAAATCCTGTGAGACACTACTGTGCCCTGTGTGTCTATATTTATCCTTGT   149
aa51_E02     -CTCTACGCTACATTTCAAGTCAATATTTACCTAAACACAAACATCAGTCCGTATTTCTGGAGAGGAGTTCAATACTGCATCTGCTTTCGATTTGCAGTGAAAATCCTGTGAGACACTACTGTGCCCTGTGTGTCTATATTTATCCTTGT   149
aa9_A08      --TCTACGCTACATTTCAAGTCAATATTTACCTAAACACAAACATCAGTCCGTATTTCTGGAGAGGAGTTCAATACTGCATCTGCTTTCGATTTGCAGTGAAAATCCTGTGAGACACTACTGTGCCCTGTGTGTCTATATTTATCCTTGT   148
aa39_D02     -CTCTACGCTACATTTCAAGTCAATATTTACCTAAACACAAACATCAGTCCGTATTTCTGGAGAGGAGTTCAATACTGCATCTGCTTTCGATTTGCAGTGAAAATCCTGTGAGACACTACTGTGCCCTGTGTGTCTATATTTATCCTTGT   149
aa41_D04     TCTCTACGCTACATTTCAAGTCAATATTTACCTAAACACAAACATCAGTCCGTATTTCTGGAGAGGAGTTCAATACTGCATCTGCTTTCGATTTGCAGTGAAAATCCTGTGAGACACTACTGTGCCCTGTGTGTCTATATTTATCCTTGT   150
aa15_B02     -CTCTACGCTACATTTCAG-TCAATATTTACCTAAACACAAACATCAGTCCGTATTTCTGGAGAGGAGTTCAATACTGCATCTGCTTTCGATTTGCAGTGAAAATCCTGTGAGACACTACTGTGCCCTGTGTGTCTATATTTATCCTTGT   148
aa52_E03     -CTCTACGCTACATTTCAAGTCAATATTTACCTAAACACAAACATCAGTCCGTATTTCTGGAGAGGAGTTCAATACTGCATCTGCTTTCGATTTGCAGTGAAAATCCTGTGAGACACTACTGTGCCCTGTGTGTCTATATTTATCCTTGT   149
aa7_A06      -CTCTACGCTACATTTCAAGTCAATATTTACCTAAACACAAACATCAGTCCGTATTTCTGGAGAGGAGTTCAATACTGCATCTGCTTTCGATTTGCAGTGAAAATCCTGTGAGACACTACTGTGCCCTGTGTGTCTATATTTATCCTTGT   149
aa23_B10     -CTCTACGCTACATTTCAAGTCAATATTTACCTAAACACAAACATCAGTCCGTATTTCTGGAGAGGAGTTCAATACTGCATCTGCTTTCGATTTGCAGTGAAAATCCTGTGAGACACTACTGTGCCCTGTGTGTCTATATTTATCCTTGT   149
aa50_E01     -CTCTACGCTACATTTCAAGTCAATATTTACCTAAACACAAACATCAGTCCGTATTTCTGGAGAGGAGTTCAATACTGCATCTGCTTTCGATTTGCAGTGAAAATCCTGTGAGACACTACTGTGCCCTGTGTGTCTATATTTATCCTTGT   149
             1.......10........20........30........40........50........60........70........80........90.......100.......110.......120.......130.......140.......150
```

CLUSTAL 2.1 MULTIPLE SEQUENCE ALIGNMENT

File: /Users/isabellaloughland/Google Drive/FrankSeebacher3/AA_alignment_3.ps   Date: Mon Feb 26 15:48:34 2018


```
            ************************************************************************************************************************************
aa12_A11    TTTGATAGCATGTGTTCTTCTATACTAGGAGAAGAAGGTCCGCCTTGTTCAAATGAGTGACACAATGGACCACCAATCAGCAGTGGGCTCTCAGGAGGATGAACCAGCCATGATTCTTCCTACCATACCTATGGGG--GGGGGGAAGAAA   296
aa21_B08    TTTGATAGCATGTGTTCTTCTATACTAGGAGAAGAAGGTCCGCCTTGTTCAAATGAGTGACACAATGGACCACCAATCAGCAGTGGGCTCTCAGGAGGATGAACCAGCCATGATTCTTCCTACCATACCTATGGGA--GGCGGAAAGAAT   297
aa24_B11    TTTGATAGCATGTGTTCTTCTATACTAGGAGAAGAAGGTCCGCCTTGTTCAAATGAGTGACACAATGGACCACCAATCAGCAGTGGGCTCTCAGGAGGATGAACCAGCCATGATTCTTCCTACCATACCTATTGGA--AGGAGGGAGGAA   297
aa11_A10    TTTGATAGCATGTGTTCTTCTATACTAGGAGAAGAAGGTCCGCCTTGTTCAAATGAGTGACACAATGGACCACCAATCAGCAGTGGGCTCTCAGGAGGATGAACCAGCCATGATTCTTCCTACCATACCTATTGGA--GGCAGTAGGGAT   298
aa25_B12    TTTGATAGCATGTGTTCTTCTATACTAGGAGAAGAAGGTCCGCCTTGTTCAAATGAGTGACACAATGGACCACCAATCAGCAGTGGGCTCTCAGGAGGATGAACCAGCCATGATTCTTCCTACCATACCTATTGGA--GGCAGTAGGGAT   296
aa13_A12    TTTGATAGCATGTGTTCTTCTATACTAGGAGAAGAAGGTCCGCCTTGTTCAAATGAGTGACACAATGGACCACCAATCAGCAGTGGGCTCTCAGGAGGATGAACCAGCCATGATTCTTCCTACCATACCTATTGGA--GGCAGTAGGGAT   295
aa38_D01    TTTGATAGCATGTGTTCTTCTATACTAGGAGAAGAAGGTCCGCCTTGTTCAAATGAGTGACACAATGGACCACCAATCAGCAGTGGGCTCTCAGGAGGATGAACCAGCCATGATTCTTCCTACCATACCTATTGGA--GGCAGTAGGGAT   297
aa45_D08    TTTGATAGCATGTGTTCTTCTATACTAGGAGAAGAAGGTCCGCCTTGTTCAAATGAGTGACACAATGGACCACCAATCAGCAGTGGGCTCTCAGGAGGATGAACCAGCCATGATTCTTCCTACCATACCTATTGGA--GGCAGTAGGGAT   296
aa48_D11    TTTGATAGCATGTGTTCTTCTATACTAGGAGAAGAAGGTCCGCCTTGTTCAAATGAGTGACACAATGGACCACCAATCAGCAGTGGGCTCTCAGGAGGATGAACCAGCCATGATTCTTCCTACCATACCTATTGGA--GGCAGTAGGGAT   297
aa31_C06    TTTGATAGCATGTGTTCTTCTATACTAGGAGAAGAAGGTCCGCCTTGTTCAAATGAGTGACACAATGGACCACCAATCAGCAGTGGGCTCTCAGGAGGATGAACCAGCCATGATTCTTCCTACCATACCTATTGGA--GGCAGTAGGGAT   298
aa49_D12    TTTGATAGCATGTGTTCTTCTATACTAGGAGAAGAAGGTCCGCCTTGTTCAAATGAGTGACACAATGGACCACCAATCAGCAGTGGGCTCTCAGGAGGATGAACCAGCCATGATTCTTCCTACCATACCTATTGGA--GGCAGTAGGGAT   297
aa10_A09    TTTGATAGCATGTGTTCTTCTATACTAGGAGAAGAAGGTCCGCCTTGTTCAAATGAGTGACACAATGGACCACCAATCAGCAGTGGGCTCTCAGGAGGATGAACCAGCCATGATTCTTCCTACCATACCTATTGGA--GGCAGTAGGGAT   296
aa18_B05    TTTGATAGCATGTGTTCTTCTATACTAGGAGAAGAAGGTCCGCCTTGTTCAAATGAGTGACACAATGGACCACCAATCAGCAGTGGGCTCTCAGGAGGATGAACCAGCCATGATTCTTCCTACCATACCTATTGGA--GGCAGTAAGGAT   297
aa22_B09    TTTGATAGCATGTGTTCTTCTATACTAGGAGAAGAAGGTCCGCCTTGTTCAAATGAGTGACACAATGGACCACCAATCAGCAGTGGGCTCTCAGGAGGATGAACCAGCCATGATTCTTCCTACCATACCTATTGGA--GGCAGTAGGGAT   296
aa8_A07     TTTGATAGCATGTGTTCTTCTATACTAGGAGAAGAAGGTCCGCCTTGTTCAAATGAGTGACACAATGGACCACCAATCAGCAGTGGGCTCTCAGGAGGATGAACCAGCCATGATTCTTCCTACCATACCTATTGGA--GGCAGTAGGGAT   296
aa17_B04    TTTGATAGCATGTGTTCTTCTATACTAGGAGAAGAAGGTCCGCCTTGTTCAAATGAGTGACACAATGGACCACCAATCAGCAGTGGGCTCTCAGGAGGATGAACCAGCCATGATTCTTCCTACCATACCTATTGGA--GGCAGTAGGGAT   297
aa56_E07    TTTGATAGCATGTGTTCTTCTATACTAGGAGAAGAAGGTCCGCCTTGTTCAAATGAGTGACACAATGGACCACCAATCAGCAGTGGGCTCTCAGGAGGATGAACCAGCCATGATTCTTCCTACCATACCTATTGGA--GGCAGTAGGGAT   297
aa54_E05    TTTGATAGCATGTGTTCTTCTATACTAGGAGAAGAAGGTCCGCCTTGTTCAAATGAGTGACACAATGGACCACCAATCAGCAGTGGGCTCTCAGGAGGATGAACCAGCCATGATTCTTCCTACCATACCTATTGGA--GGCAGTAGGGAT   297
aa16_B03    TTTGATAGCATGTGTTCTTCTATACTAGGAGAAGAAGGTCCGCCTTGTTCAAATGAGTGACACAATGGACCACCAATCAGCAGTGGGCTCTCAGGAGGATGAACCAGCCATGATTCTTCCTACCATACCTATTGGA--GGCAGTAGGGAT   297
aa53_E04    TTTGATAGCATGTGTTCTTCTATACTAGGAGAAGAAGGTCCGCCTTGTTCAAATGAGTGACACAATGGACCACCAATCAGCAGTGGGCTCTCAGGAGGATGAACCAGCCATGATTCTTCCTACCATACCTATTGGA--GGCAGTAGGGAT   296
aa32_C07    TTTGATAGCATGTGTTCTTCTATACTAGGAGAAGAAGGTCCGCCTTGTTCAAATGAGTGACACAATGGACCACCAATCAGCAGTGGGCTCTCAGGAGGATGAACCAGCCATGATTCTTCCTACCATACCTATTGGA--GGCAGTAGGGAT   297
aa26_C01    TTTGATAGCATGTGTTCTTCTATACTAGGAGAAGAAGGTCCGCCTTGTTCAAATGAGTGACACAATGGACCACCAATCAGCAGTGGGCTCTCAGGAGGATGAACCAGCCATGATTCTTCCTACCATACCTATTGGA--GGCAGTAGGGAT   297
aa34_C09    TTTGATAGCATGTGTTCTTCTATACTAGGAGAAGAAGGTCCGCCTTGTTCAAATGAGTGACACAATGGACCACCAATCAGCAGTGGGCTCTCAGGAGGATGAACCAGCCATGATTCTTCCTACCATACCTATTGGA--GGCAGTAGGGAT   297
aa6_A05     TTTGATAGCATGTGTTCTTCTATACTAGGAGAAGAAGGTCCGCCTTGTTCAAATGAGTGACACAATGGACCACCAATCAGCAGTGGGCTCTCAGGAGGATGAACCAGCCATGATTCTTCCTACCATACCTATTGGA--GGCAGTAGGGAT   296
ncaa2_A02   TTTGATAGCATGTGTTCTTCTATACTAGGAGAAGAAGGTCCGCCTTGTTCAAATGAGTGACACAATGGACCACCAATCAGCAGTGGGCTCTCAGGAGGATGAACCAGCCATGATTCTTCCTACCATACCTATTGGA--GGCAGTAGG[illegible]T   298
aa55_E06    TTTGATAGCATGTGTTCTTCTATACTAGGAGAAGAAGGTCCGCCTTGTTCAAATGAGTGACACAATGGACCACCAATCAGCAGTGGGCTCTCAGGAGGATGAACCAGCCATGATTCTTCCTACCATACCTATTGGG--GGCTAAAGGGAT   298
aa42_D05    TTTGATAGCATGTGTTCTTCTATACTAGGAGAAGAAGGTCCGCCTTGTTCAAATGAGTGACACAATGGACCACCAATCAGCAGTGGGCTCTCAGGAGGATGAACCAGCCATGATTCTTCCTACCATACCTATTGGA--GGCAGTAGGGAT   298
NC_AA_4_D10 TTTGATAGCATGTGTTCTTCTATACTAGGAGAAGAAGGTCCGCCTTGTTCAAATGAGTGACACAATGGACCACCAATCAGCAGTGGGCTCTCAGGAGGATGAACCAGCCATGATTCTTCCTACCATACCTATTGGA--GGCAGTAGGGAT   296
aa37_C12    TTTGATAGCATGTGTTCTTCTATACTAGGAGAAGAAGGTCCGCCTTGTTCAAATGAGTGACACAATGGACCACCAATCAGCAGTGGGCTCTCAGGAGGATGAACCAGCCATGATTCTTCCTACCATACCTATTGGA--GGCAGTAAGGAT   297
aa19_B06    TTTGATAGCATGTGTTCTTCTATACTAGGAGAAGAAGGTCCGCCTTGTTCAAATGAGTGACACAATGGACCACCAATCAGCAGTGGGCTCTCAGGAGGATGAACCAGCCATGATTCTTCCTACCATACCTATTGGA--GGCAGTAGGGAT   297
aa33_C08    TTTGATAGCATGTGTTCTTCTATACTAGGAGAAGAAGGTCCGCCTTGTTCAAATGAGTGACACAATGGACCACCAATCAGCAGTGGGCTCTCAGGAGGATGAACCAGCCATGATTCTTCCTACCATACCTATTGGA--GGCAGTAGGGAT   297
NC_AA_1_A10 TTTGATAGCATGTGTTCTTCTATACTAGGAGAAGAAGGTCCGCCTTGTTCAAATGAGTGACACAATGGACCACCAATCAGCAGTGGGCTCTCAGGAGGATGAACCAGCCATGATTCTTCCTACCATACCTATTGGA--GGCAGTAGGGAT   297
aa28_C03    TTTGATAGCATGTGTTCTTCTATACTAGGAGAAGAAGGTCCGCCTTGTTCAAATGAGTGACACAATGGACCACCAATCAGCAGTGGGCTCTCAGGAGGATGAACCAGCCATGATTCTTCCTACCATACCTATTGGA--GGCAGTAAGGAA   297
aa29_C04    TTTGATAGCATGTGTTCTTCTATACTAGGAGAAGAAGGTCCGCCTTGTTCAAATGAGTGACACAATGGACCACCAATCAGCAGTGGGCTCTCAGGAGGATGAACCAGCCATGATTCTTCCTACCATACCTATTGGA--GGCAGTAAGGAA   298
aa30_C05    TTTGATAGCATGTGTTCTTCTATACTAGGAGAAGAAGGTCCGCCTTGTTCAAATGAGTGACACAATGGACCACCAATCAGCAGTGGGCTCTCAGGAGGATGAACCAGCCATGATTCTTCCTACCATACCTATTGGA--GGCAGTAGGGAA   296
aa43_D06    TTTGATAGCATGTGTTCTTCTATACTAGGAGAAGAAGGTCCGCCTTGTTCAAATGAGTGACACAATGGACCACCAATCAGCAGTGGGCTCTCAGGAGGATGAACCAGCCATGATTCTTCCTACCATACCTATTGGA--GGAAAAAAGGAT   297
NC_AA_5_E10 TTTGATAGCATGTGTTCTTCTATACTAGGAGAAGAAGGTCCGCCTTGTTCAAATGAGTGACACAATGGACCACCAATCAGCAGTGGGCTCTCAGGAGGATGAACCAGCCATGATTCTTCCTACCATACCTATTGGA--GGCAGTAGGGAT   297
aa57_E08    TTTGATAGCATGTGTTCTTCTATACTAGGAGAAGAAGGTCCGCCTTGTTCAAATGAGTGACACAATGGACCACCAATCAGCAGTGGGCTCTCAGGAGGATGAACCAGCCATGATTCTTCCTACCATACCTATTGTG--GGTATGTGGGAT   297
NC_AA_2_B10 TTTGATAGCATGTGTTCTTCTATACTAGGAGAAGAAGGTCCGCCTTGTTCAAATGAGTGACACAATGGACCACCAATCAGCAGTGGGCTCTCAGGAGGATGAACCAGCCATGATTCTTCCTACCATACCTATTGGA--GGCAGTAGGGAT   297
aa40_D03    TTTGATAGCATGTGTTCTTCTATACTAGGAGAAGAAGGTCCGCCTTGTTCAAATGAGTGACACAATGGACCACCAATCAGCAGTGGGCTCTCAGGAGGATGAACCAGCCATGATTCTTCCTACCATACCTATTGGA--GGCAGTAGGGAT   297
aa44_D07    TTTGATAGCATGTGTTCTTCTATACTAGGAGAAGAAGGTCCGCCTTGTTCAAATGAGTGACACAATGGACCACCAATCAGCAGTGGGCTCTCAGGAGGATGAACCAGCCATGATTCTTCCTACCATACCTAT-------GCAGTAGGGAT   292
aa47_D10    TTTGATAGCATGTGTTCTTCTATACTAGGAGAAGAAGGTCCGCCTTGTTCAAATGAGTGACACAATGGACCACCAATCAGCAGTGGGCTCTCAGGAGGATGAACCAGCCATGATTCTTCCTACCATACCTATTGGA--GGCAGAAGGGAA   297
aa20_B07    TTTGATAGCATGTGTTCTTCTATACTAGGAGAAGAAGGTCCGCCTTGTTCAAATGAGTGACACAATGGACCACCAATCAGCAGTGGGCTCTCAGGAGGATGAACCAGCCATGATTCTTCCTACCATACCTATTG-----GCGGTAGGGAT   294
aa46_D09    TTTGATAGCATGTGTTCTTCTATACTAGGAGAAGAAGGTCCGCCTTGTTCAAATGAGTGACACAATGGACCACCAATCAGCAGTGGGCTCTCAGGAGGATGAACCAGCCATGATTCTTCCTACCATACCTATTGGA--GGGGGAAGGGAA   298
aa14_B01    TTTGATAGCATGTGTTCTTCTATACTAGGAGAAGAAGGTCCGCCTTGTTCAAATGAGTGACACAATGGACCACCAATCAGCAGTGGGCTCTCAGGAGGATGAACCAGCCATGATTCTTCCTACCATACCTATTGGA--GGCAGTAGGGAT   296
aa35_C10    TTTGATAGCATGTGTTCTTCTATACTAGGAGAAGAAGGTCCGCCTTGTTCAAATGAGTGACACAATGGACCACCAATCAGCAGTGGGCTCTCAGGAGGATGAACCAGCCATGATTCTTCCTACCATACCTATGGGG--AGTAGAAGGGAT   296
aa36_C11    TTTGATAGCATGTGTTCTTCTATACTAGGAGAAGAAGGTCCGCCTTGTTCAAATGAGTGACACAATGGACCACCAATCAGCAGTGGGCTCTCAGGAGGATGAACCAGCCATGATTCTTCCTACCATACCTATTGTA--GGCAGAAGGGAA   298
aa58_E09    TTTGATAGCATGTGTTCTTCTATACTAGGAGAAGAAGGTCCGCCTTGTTCAAATGAGTGACACAATGGACCACCAATCAGCAGTGGGCTCTCAGGAGGATGAACCAGCCATGATTCTTCCTACCATACCTATGGGG--GGTAGAG-----   292
aa51_E02    TTTGATAGCATGTGTTCTTCTATACTAGGAGAAGAAGGTCCGCCTTGTTCAAATGAGTGACACAATGGACCACCAATCAGCAGTGGGCTCTCAGGAGGATGAACCAGCCATGATTCTTCCTACCATACCTATTGGA--GGCAGGGGGGAA   297
aa9_A08     TTTGATAGCATGTGTTCTTCTATACTAGGAGAAGAAGGTCCGCCTTGTTCAAATGAGTGACACAATGGACCACCAATCAGCAGTGGGCTCTCAGGAGGATGAACCAGCCATGATTCTTCCTACCATACCTATT-----------AGGGAC   287
aa39_D02    TTTGATAGCATGTGTTCTTCTATACTAGGAGAAGAAGGTCCGCCTTGTTCAAATGAGTGACACAATGGACCACCAATCAGCAGTGGGCTCTCAGGAGGATGAACCAGCCATGATTCTTCCTACCATACCTATAGT---------AGGGAT   290
aa41_D04    TTTGATAGCATGTGTTCTTCTATACTAGGAGAAGAAGGTCCGCCTTGTTCAAATGAGTGACACAATGGACCACCAATCAGCAGTGGGCTCTCAGGAGGATGAACCAGCCATGATTCTTCCTACCATACCTAT-GGA--GGGAATGGGGAA   297
aa15_B02    TTTGATAGCATGTGTTCTTCTATACTAGGAGAAGAAGGTCCGCCTTGTTCAAATGAGTGACACAATGGACCACCAATCAGCAGTGGGCTCTCAGGAGGATGAACCAGCCATGATTCTTCCTACCATACCTATGGTG--GGGAAGGAAGAA   296
aa52_E03    TTTGATAGCATGTGTTCTTCTATACTAGGAGAAGAAGGTCCGCCTTGTTCAAATGAGTGACACAATGGACCACCAATCAGCAGTGGGCTCTCAGGAGGATGAACCAGCCATGATTCTTCCTACCATACCTATTGGG--GGGGAAGGAAAA   297
aa7_A06     TTTGATAGCATGTGTTCTTCTATACTAGGAGAAGAAGGTCCGCCTTGTTCAAATGAGTGACACAATGGACCACCAATCAGCAGTGGGCTCTCAGGAGGATGAACCAGCCATGATTCTTCCTACCATACCTATTGGG--GGAGAAGGAGAT   297
aa23_B10    TTTGATAGCATGTGTTCTTCTATACTAGGAGAAGAAGGTCCGCCTTGTTCAAATGAGTGACACAATGGACCACCAATCAGCAGTGGGCTCTCAGGAGGATGAACCAGCCATGATTCTTCCTACCATACCTAT-GGG--AGTAAGGAGGAA   296
aa50_E01    TTTGATAGCATGTGTTCTTCTATACTAGGAGAAGAAGGTCCGCCTTGTTCAAATGAGTGACACAATGGACCACCAATCAGCAGTGGGCTCTCAGGAGGATGAACCAGCCATGATTCTTCCTACCATACCTATTGGG--GGCAAGGAGAAA   297
            .......160.......170.......180.......190.......200.......210.......220.......230.......240.......250.......260.......270.......280.......290.......300
```

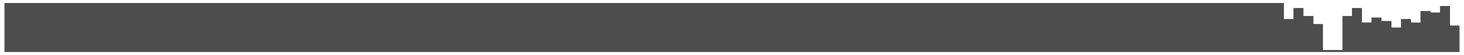

CLUSTAL 2.1 MULTIPLE SEQUENCE ALIGNMENT

File: /Users/isabellaloughland/Google Drive/FrankSeebacher3/AA_alignment_3.ps   Date: Mon Feb 26 15:48:34 2018


```
                    **           * *   **                      ***
aa12_A11    AAGGGAGGAAAAAATTTTGTTTTTTTTTTTTGCGG--GGGGGG----TCTTTTCTTTTTTTTT-TAATGTTTTTTTAATAGGGG---GGGTATTGTTGTTG-AGGGGGGGGACCCAAAAAAAACACCACCCCAGCCAAACCAACTTCTTT   435
aa21_B08    AAAGCAAGAAAATGTTTTGTTTTTTTTCTTTCCGG--GGGGCG----GCTTTACTGATTTTGT-TATTGTTATTTTAATGGGGA---GGGTATTGTGGTTG-TGGTGGGGTCTCAAAAAAAAACCCCGCCCCGGCCAAACCA-CTTCCTC   435
aa24_B11    AAAGGAGGGAAAAAATTTG---TTTTTTTTTCTTG--CGGGCGGCG-GTTCTGATGAATTTTT-TTATGTTTTTTTAATATTGG---GGGAAATGTTGTTA-TCGTGGGGAAATCAAAAAAAAAAAAAATCCTGCCAAATCAACATATTT   436
aa11_A10    AAAGCAGGCAAAAAATTTGTAAGTTTTCTTTCCTG--CGGGCG----GCTTTTCTGAATTTTT-TAAGGCAATTTTTAAAGCCT---GGGAAACCTGGTTA-ACTTGGGGAACCCATCAAATACACCAATCCGGCCAAATCAACAGCATT   437
aa25_B12    AAAGCAGGCAAAAAATTTGTAAGTTTTCTTTCCTG--CGGGCG----GCTTTTCTGAATTTTT-TAAGGCTATTTTTATATCCT---GGGAAATCTGGTTA-ACTTTGGGAACTCATCAAAAACGCCAACCCGGCCAAATCAACGGCATT   435
aa13_A12    AAAGCAGGCAAAAAATTTGTAAGTTTTCTTTCCTG--CGGGCG----GCTTTTCTGAATTTTT-TAAGGTTATTTTTAAAGCCA---GGGAAATCTTGTTG-ACATTGGGAACCCATCAAAAACACCAACCCGGCCAAATCAACACCATT   434
aa38_D01    AAAGCAGGCAAAAAATTTGTAAGTTTTCTTTCCTG--CGGGCG----GCTTTTCTGAATTTTT-TAAGGCTATTTTTATAGTCA---GGGAAATCTTGTTG-ACTTTGGGAACTCATCAAATACACCAATCCGGCCAAATCAACAGCATT   436
aa45_D08    AAAGCAGGCAAAAAATTTGTAAGTTTTCTTTCCTG--CGGGCG----GCTTTTCTGAATTTTT-TAAGGCTATTTTTATAGTCA---TGGAAATCTTGTTG-ACTTTGTGAACTCATCAAAAACACCAATCCGGCCAAATCAACACCATT   435
aa48_D11    AAAGCAGGCAAAAAATTTGTAAGTTTTCTTTCTTG--CGGGCG----GCTTTTCTGAATTTTT-TAAGGCTATTTTTATAGTCA---GGGAAATCTTGTTG-ACTTTGGGAACTCATCAAAAACACCAACCCGGCCAAATCAACACCTTT   436
aa31_C06    AAAGCAGGCAAAAAATTTGTAAGTTTTCTTTCCTG--CGGGCG----GCTTTTCTGAATTTTT-TATGGCTTTTTTTATAGCCA---GGGAAATCTTGTTG-ACTTTGGGAACCCATCAAATACACCAACCCGGCCAAACCAACACCATT   437
aa49_D12    AAAGCAGGCAAAAAATTTGTAAGTTTTCTTTCCTG--CGGGCG----GCTTTTCTGAATTTTT-TATGGTTATTTTAATATTCT---GGGAAATCTTGTTG-ACTTTGGGAACTCATCAAATACAACAATCCGGCCAAATCAACACCATT   436
aa10_A09    AAAGCAGGCAAAAAATTTGTAAGTTTTCTTTCTTG--CGGGCG----GCTTTTCTGAATTTTT-TATGGCTTTTTTAATATCCT---GGGAATTCTGGTTG-ACTTTGGGAACCCATCAAAAACACCATCCCGGCCAAACCAACACCATT   435
aa18_B05    AAAGCAGGCAAAAAATTTGTTTGTTTTCTTTCCTG--CGGGCG----GCTTTTCTGAATTTTT-TATGGTTATTTTAATGGCAA---GGGAATTCTGGTTG-ACTTGGGGACCTCATCAAAAACCCCAACCCGGCCAAACCAACACCTTT   436
aa22_B09    AAAGCAGGCAAAAAATTTGTAAGTTTTCTTTCCTG--CGGGCG----GCTTTTCTGAATTTTT-TATGGTTATTTTTATAGCCA---GGGAAATCTGGTTG-ACTTGGGGAACCCATCAAAAACACCATCCCGGCCAAACCAACACCTTT   435
aa8_A07     AAAGCAGGCAAAAAATTTGTAAGTTTTCTTTCCTG--CGGGCG----GCTTTTCTGAATTTTT-TATGGTTTTTTTTAAATTCT---GGGAAATCTTGTTA-ACTTTGGGAACCCACCAAAAAAACCATCCCGGCCAAACCAACACCTTT   435
aa17_B04    AAAGCAGGCAAAAAATTTGTAAGTTTTCTTTCCTG--CGGGCG----GCTTTTCTGAATTTTT-AATGGCAATTTTAAAATCCT---GGGAAACCTGGTGA-ACTTGGGGAATTCTCCAAAAACACAATCCCGGCCAAACCACCACCATT   436
aa56_E07    AAAGCAGGCAAAAAATTTGTAAGTTTTCTTTCCTG--CGGGCG----GCTTTTCTGAATTTTT-TATGGCTATTTTAAAATCCT---GGGAAACCTGGTGA-ACTTGGGGAATTCTCCAAAAACACAATCCCGGCCAAACCACCACCTTT   436
aa54_E05    AAAGCAGGCAAAAAATTTGTAAGTTTTCTTTCCTG--CGGGCG----GCTTTTCTGAATTTTT-TATGGCAATTTTAAAATCCT---GGGAAACCTGGTTA-ACTTGGGGAATCCTCCAAAAACACAATCCCGGCCAAACCACCACATTT   436
aa16_B03    AAAGCAGGCAAAAAATTTGTAAGTTTTCTTTCCTG--CGGGCG----GCTTTTCTGAATTTTT-AAAGGCAATTTTAAAATCCT---GGGAAACCTGGTTA-ACTTTGGGAATCCACCAAAAACACCATCCCGGCCAAACCAACACCTTT   436
aa53_E04    AAAGCAGGCAAAAAATTTGTAAGTTTTCTTTCCTG--CGGGCG----GCTTTTCTGAATTTTT-AATGGCATTTTTAAAATCCT---GGGAAACCTGGTGA-ACTTTGGGAATCCTCCAAAAACACAATCCCGGCCAATCCACCACATTT   435
aa32_C07    AAAGCAGGCAAAAAATTTGTAAGTTTTCTTTCTTG--CGGGCG----GCTTTTCTGAATTTTT-AAAGGTTTTTTTAAAATCCT---GGGAATCCTGGTTA-ACTTGGGGAATCCACCAAAAACGCCATCCCGGCCAATCCACAACCTTT   436
aa26_C01    AAAGCAGGCAAAAAATTTGTAAGTTTTCTTTCTTG--CGGGCG----GCTTTTCTGAATTTTT-TATGGTTATTTTAATATCCT---GGGAATTCTGGTTA-ACTTGGGGAACCCACCAAAAACACAATCCCGGCCAAACCAAAACCTTT   436
aa34_C09    AAAGCAGGCAAAAAATTTGTAAGTTTTCTTTCTTG--CGGGCG----GCTTTTCTGAATTTTT-AATGGTTTTTTTAAAATCCT---GGGAATCCTGGTGA-ACTTGGGGAATCCTCAAAAAACACAATCCCGGCAAAACCACAACATTT   436
aa6_A05     AAAGCAGGCAAAAAATTTGTAAGTTTTCTTTCCTG--CGGGCG----GCTTTTCTGAATTTTT-TATGGTTTTTTTAAAATCCT---GGGAAACCTGGTTA-ACTTGGGGAACCCACCAAATCCACCATCCCGGCCAAACCACAATCATT   435
ncaa2_A02   AAAGCAGGCAAAAAATTTGTAAGTTTTCTTTCCTG--CGGGCG----GCTTTTCTGAATTTTT---------------------------------------------------------------------------------------   355
aa55_E06    AAAGCAAGAAAAAAATTTGAAATTTTTCTTTCTTG--CGGGCG----GCTTTTCCGAATTTTT-TAAGGTTTTTTTTATATTTT---TGGAAATGTGATTC-ATTTGGGGAATCCGCAAAAACCACAAACCCGGCCCATCCACCATCATT   437
aa42_D05    AAAGCAGGCAAAAAATTTGTAATTTTTCTTTCTTG--CGGGCG----GCTTTTCTGAATTTTT-TATGGTTTTTTTATGGTCAT---GGGAATCCTGGTGA-AATTTGGGAACCCTCAAAAAACACAATCCTGCCCAAACCACCCCATTT   437
NC_AA_4_D10 AAAGCAGGCAAAAAATTTGTAAGTTTTCTTTCCTG--CGGGCG----GCTTTTCTGAATTTTT---------------------------------------------------------------------------------------   353
aa37_C12    AAAGCAGGCAAAAAATTTGTAATTTTTCTTTCCTG--CGGGCG----GCTTTTCTGAATTTTT-TATTGTTTTTTTTAAATCCT---GGGGATTCTGGTGA-ACATGGGGAATCCACAAAAAACACAAATCCGGCCAAACCAACCCCATT   436
aa19_B06    AAAGCAGGCAAAAAATTTGTAATTTTTCTTTCTTG--CGGGCG----GCTTTTCTGAATTTTT-TATGGTTTTTTTAAAAGCCA----GGGAATCTTGGTGA-ACTTGGGGAACCCAAAAAAAAAACCATCCCGGCCAAACCACATTATT   435
aa33_C08    AAAGCAGGCAAAAAATTTGTATTTTTTCTTTCCTG--CGGGCG----GCTTTTCTGAATTTTT-AATGCTTTTTTTATAAGCGT----GGGAATCTTGGTGA-CTTTGGGGACCCAACAAAAACAACCATCCCGCCAAATCCACATTCTT   435
NC_AA_1_A10 AAAGCAGGCAAAAAATTTGTAAGTTTTCTTTCCTG--CGGGCG----GCTTTTCTGAATTTTT---------------------------------------------------------------------------------------   354
aa28_C03    AAAACAAGCAAAAAATTTGTTTGTTTTCTTTCCTG--GGGGGG----GCTTTTCTGAATTTTT-TATTGTTTTTTTTAAAACCG---GGGAAACTTTGTGG-ACTTTGGGGATCCACCAAAAAAACCAACCCGGCCAAATCACCACATTT   436
aa29_C04    AAAACAAGCAAAAAATTTGTTTGTTTTCTTTCCTG--GGGGGG----GCTTTTCTGAATTTTT-TATTGTTTTTTTTAAAACCG---GGGAAACTTTGTGG-ACTTTGGGGATCCACCAAAAAAACCAACCCGGCCAAATCACCACATTT   437
aa30_C05    AAAGCAGGCAAAAAATTTGTAATTTTTCTTTCCTG--GGGGCG----GCTTTTCTGAATTTTT-TAGGGTTTTTTTTAAAGCCG---GGGAAATCTTGTTG-ACTTTGGGAACCCCACAAAAAACCAAACCCGGCCAAATCAACACTTTT   435
aa43_D06    AAAACAAGCAAAAAATTTGTAAGTTTTCTTTCCTG--TTGGCG----GCTTTTCTGAATTTTT-TTTGGTTTTTTTAATATCTT---GGGGAAACTTGTTG-AACTTGTGAAATCACCAAAACCAGCAATCCTGCCAAATCACCCCCATT   436
NC_AA_5_E10 AAAGCAGGCAAAAAATTTGTAAGTTTTCTTTCCTG--CGGGCG----GCTTTTCTGAATTTTT---------------------------------------------------------------------------------------   354
aa57_E08    TAAGCAAGCAAAAAATTTGTATTTTTTTTTTCCGG--CGGGTG---GGATTTTTTATTTTTTT-TATG-CTATTTTTATAATCC---TGGGAATCTTGTTG-AAATGGTGGAGTCAACCAAATCGACAATCCTGCCAAAACAACCACATT   436
NC_AA_2_B10 AAAGCAGGCAAAAAATTTGTAAGTTTTCTTTCCTG--CGGGCG----GCTTTTCTGAATTTTT-TATGGCTATTTT--------------------------------------------------------------------------   366
aa40_D03    AAAGCAGGCAAAAAATTTGTAAGTTTTCTTTCCTG--CGGGCG----GCTTTTCTGAATTTTT-TAATGCTATTTTTATAGTCA---TGGGAATCTTGTTG-ACTTTGTGGACTCATCAAATACAGCAATCCTGCCAAATCAACAGCATT   436
aa44_D07    AAAGCAGGCAAAAAATTTGTAAGTTTTCTTTCCTG--CGGGCG----GCTTTTCTGAATTTTT-TAATGCTATTTTTATAGTCA---TGGGAATCTTGTTG-ACATTGTGGACTCATCAAATACAGCAATCCTGCCAAATCAACACCATT   431
aa47_D10    AAAGCAGGAAAAAAATTTGTAATTTTTCTTTCTTG--CGGGCG----GCTTTTCTGAATTTTT--AATGCTATTTTTATAGTCA---TGGGAATCTTGTTG-ACATTGTGGACTCATCAAATACAGCAATCCTGCCAAATCCACAGCATT   435
aa20_B07    AAAGCAGGCAAAAAATTTGTAATTTTTCTTTCTTG--CGGGCG----GCTTTTCTGAATTTTT--AATGCTATTTTTATAGTCA---TGGGAATCTTGTTG-ACATTGTGGACTCATCAAAAACAACAATCCTGCCAAATCCACAACATT   432
aa46_D09    AAAGAAGGCAAAAAATTTTTATGTTTTTTTTCTTG--CGGGGG----GTTTTTCT-AATTTTT-TTATGGTATTTTTATTGTCA---TGGGAATCTTGTTG-ACATTGTGGACTCATCAAAAACAAAAATCCCGCCAAATCCACAGCATT   436
aa14_B01    AAAGCAGGCAAAAAATTTGTAAGTTTTCTTTCTTG--CGGGCG----GCTTTTCTGAATTTTT--TATGGTATTTTTATAATCC---TGGGAATCTTGGTG-AAATTGGGGAATCCACAAAAACAACAATCCTGCCCAATCCACAGCATT   434
aa35_C10    AGGGAAAACAAAGGTATGGTTTTTGTCCTTTCCCGTTCTTGCCTGT-GCTTTTTTTGATTTTG-TAATGTTATTTTTAAGGTGA---GGGTAATCAGGTTG-ACGATGCGAACCAAAAAAATACCCCATCCCAGCCAAATCCATTACTTT   440
aa36_C11    AAGGCAAGAAAAAAATTTGTTTCTTTTCTTTGCGG--CGGGCG----GCTTTTCTGATTTTTG-TAATGTTTTTTTTAAAGTCA---GGCGAGTTGTGTTG-ATGGTGTGAACACATAAAATAAACCAGTCCAGTCAAATCAATTTCATC   437
aa58_E09    AAAGCAAAGGAAAAAAAATGTATTTTTTCTTTCTCGTGCGGGGG----GCTTCTCTGATTTTT--TTATGATATTTTTATTGTCA---GGGAAGTTGAGTTG-ATTTTGTGAACTCATCAAACAAAACAACCCAGCCAACCCCACTCCATT   432
aa51_E02    AAAGGAGGAAAAAATTTTTTTTGTTTTCTTTCTTG--CGGGCG---TTTTTTCTGATTTTTT--TATGCTTTTTTTATACTCG---TGGAAATCTTGTTT-ACATTGGGTACTCATCAAATACAAGAATCCTGCCAAATCCACAGATTT   435
aa9_A08     AAAGCAGGCAAAAAACAAGAAAATTTTTTTTCTTT--TTTCTT----GCGTG-CGGGATTTTT-TAATGTTTTTTTAATGTTATT-TT--AATCGTGGTG-AAATTGTTGATTCTGCAGAAAACACAAACCAGCCAAATCCCCAGCCTT   425
aa39_D02    AAAGGATGAAAAAAATTTGAAATTTTTCTTTTTTG--CTTCCG----GCTTTTCGGATTTTTT-AAATGTTAATTGTATTTTCATG-GG--AATCGGGATG-ATGTTGGGGATTCGGCACACACAGAAATCCTGCCCCATCCAAAACCTT   429
aa41_D04    AAACGACGAAAAAAAAATTATAAATTTTCTTTTTTG--GGGGCG----GGTTTTCTTAATTTTT-AATTTTTTTTTTTTGCACGC---GGG-TTTTTTGTTT-TTTTTGGGGACTCATCAAAAACAGCAACCCCGCCAAATCCACAATCTT   435
aa15_B02    AAAAGAAGAAAAAAATTTTTTTGTTTTCTTTGGTG--GCGGCGG---TTTTTTTTTTTTTTTT-TGATGTTTTTTTAATAGTGA---TGGTTATGTTGATG-TTGTGGTGGAATCATAAAAAAATCCCATCCCGCCACAACACCAACTCC   435
aa52_E03    AAAAGGGGAAAAAATTTTTGTTTTTTTTTTTTCGGG--GGGGGGT----TTTCTGAATTTTTTT-TGCTGTTTTTATTGTCGTGG---GGGTCTTGTTGACG-TTGTGGACGACTCATAAAAAACAACCCTCCTGCCTCCACACCATTTTT   436
aa7_A06     AAAGGGAGAAAAAATTTTGTATTTTTTTTTTTGTG--GGGGTGT----CTTTTGTGATTTTTT-TGTTGTTTTTTTATTCTTGT---TATTCTTGTTGTCA-TGGTGGGCTCATCAAAAAAACCAATCCTGCAGAAAATTCAACATTTCT   436
aa23_B10    AAAAGGAAGAAAAAAGGTAGAAATTTTTCTTGCTG--GCGGCCGG---CTTGGCGGTTTTTTT-TATTGTTTTATGGATATTGT---AAAGATTGTGGAAA-TCTGGGGGAATTCAGAAAAAACACCAATCCGGCAACCCGACCATTTCT   436
aa50_E01    AAGGGAAAAAAAATTTTTAAATTTTTTTCTTGCTG--GTGGCTG----CTTTTGATTTTTTTT-AGTTGTTTTTTTAATCTTGT---GAATATTGTTGATA-TTGTGGAGTCATCATAAAAAACAATATTCCGGCATCATCAACATTTTT   436
            .......310.......320.......330.......340.......350.......360.......370.......380.......390.......400.......410.......420.......430.......440.......450
```

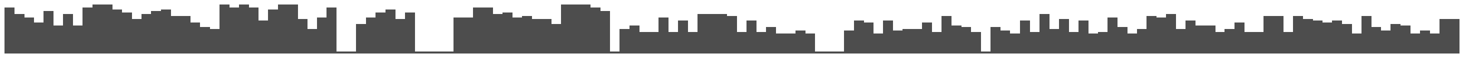

```
aa12_A11     T------------CCACAAATCACAGAAAAATG--ATCCCG---------------------------ACCACACCCCCCAGAAAGAAAATTGTTTTTT--------TTCTTGTT-----------------------------------   501
aa21_B08     T------------TCTCACAACCAAGGGAAATT--GACCTG---------------------------AACCCACCCCCCGTAGAGGAAAAAATTTTCTG-------TTCTGGTT-----------------------------------   502
aa24_B11     T------------CCTAAAATCGTCGAAAAATG--GCGTTG----------------------------ACACAACCCCCAAACATGAAAAAATGTTTTTT-------TCCTGTT-----------------------------------   502
aa11_A10     T------------CCCCAAATCACGGCAAAAGG--ATCTTG---------------------------ACCAAACTTCCCAAACGGAAAAAAGTTTCATT--------TCAGGCT-----------------------------------   503
aa25_B12     T------------CCCCAAATCACGGCAAAAGG--ATCTTG---------------------------ACCAAACTTCCCAAACGGAAAAAAGTTTCATT--------TCAGGCT-----------------------------------   501
aa13_A12     T------------CCTCAAATCACGGCAAAAGG--ACCTTG---------------------------ACCAAACATCCCAAACGGAAAAAAGTTTCATT--------TCAGGCT-----------------------------------   500
aa38_D01     T------------CCTCAAATCACGGCAAAAGG--ATCTTG---------------------------ACCAAACTTCCCAAACGGAAAAAAGTTTCATT--------TCAGGCT-----------------------------------   502
aa45_D08     T------------CCCCAAATCACTGCAAAATG--ATCTTG---------------------------ACCAAACTTCCCAAACTGAAAAAAGTTTCATT--------TCAGGCT-----------------------------------   501
aa48_D11     T------------CCTCAAATCACTGCAAAAGG--ATCTTG---------------------------ACCAAACATCCCAAACGGAAAAAAGTTTCATT--------TCAGGCT-----------------------------------   502
aa31_C06     T------------CCCCAAATCACTGCAAAAGG--ATCTTG---------------------------ACCAAACTTCCCAAACGGAAAAAAGTTTCTTT--------TCAGGCT-----------------------------------   503
aa49_D12     T------------CCTCAAATCACGGCAAAAGG--ATCTGG---------------------------ACCAGACTTCCCATACGGTAAAAAGTTTCTTT--------TCAGGTT-----------------------------------   502
aa10_A09     T------------CCCCAAATCATGGAAAAAGG--ATCTTG---------------------------ACCAAACTTCCCAAACGGAAAAAGTTTTCATT--------TCAGGTT-----------------------------------   501
aa18_B05     T------------CTCCAAACCACGGGAAAATG--ACCTGA---------------------------ACAAACCTCCCAAAACGGAAAAAATTTCCTTT--------TCAGGTT-----------------------------------   502
aa22_B09     T------------CCTCAAACCATGGCAAAAGG--ACCTGG---------------------------ACAAAACACCCAAAACGGAAAAAATTTTCTTT--------TCAGGTT-----------------------------------   501
aa8_A07      T------------CCTCAAATCATGGCAAAAGG--ACCTGG---------------------------ACCAAACTCCCAAAACGGAAAAAGTTTCCTTT--------TCAGGTT-----------------------------------   501
aa17_B04     C------------CCCCAAACCATGGCAAAAGG--ACCTGG---------------------------ACCAAACTTCCCAAACGGAAAAAGGTTCCTTT--------TCAGGTT-----------------------------------   502
aa56_E07     C------------CCCCAAACCATGGCAAAAGG--ACCTGG---------------------------ACCAAACTTCCCAAACGGAAAAAGGTTCCTTT--------TCAGGTT-----------------------------------   502
aa54_E05     T------------CCCCAAACCATGGCAAAAGG--ACCTGG---------------------------ACCAAACTCCCCAAACGGAAAAAGGTTCCTTT--------TCAGGTT-----------------------------------   502
aa16_B03     T------------CCCCAAATCATGGCAAAAGG--ACCTGG---------------------------ACCAAACTTCCCAAACGGAAAAAGTTTTCTTT--------TCAGGTT-----------------------------------   502
aa53_E04     C------------CCCCAAACCCTGGAAAATGG--ACCTGA---------------------------ACAAAACTCCCCTAATGGAAAAAGTTTCCTTT--------CCGGGTT-----------------------------------   501
aa32_C07     C------------CCCCAAACCATGGCAAATGG--ACCTGG---------------------------ACAAACCTCCCAAAATGGAAAAAGTTTCCTTT--------CCAGCTT-----------------------------------   502
aa26_C01     C------------CCCCAAACCCTGGCAAATGG--ACCTGG---------------------------ACCAAACTTCCCAAACGGAAAAAGTTTCCTTT--------TCAGGTT-----------------------------------   502
aa34_C09     C------------CCCCAAACCCTGGAAAATGG--ACCTGA---------------------------ACCAAACTCCCCAAATGGAAAAAGTTTTCTTT--------TCAGGTT-----------------------------------   502
aa6_A05      C------------CCCCAAATCATGGAAAATGG--ACCTTG---------------------------ACCAACCTCCCCTAACGGAAAAAGGTTTCTTT--------TCAGGTT-----------------------------------   501
ncaa2_A02    ------------------------------------------------------------------------------------------------------------------------------------------------------   355
aa55_E06     C------------CCCCTAACCCTGACAAATGG--ACCTTG---------------------------ACCAAGACCCCACACCGGAAAAAGTTTCCATT--------TCCGGTT-----------------------------------   503
aa42_D05     C------------CCCCAAACAACGGAAAATGA--ACCTGA---------------------------ACCAAACACCCCAAAAGGAAAAAATTTCCTTT--------TCGGGTT-----------------------------------   503
NC_AA_4_D10  ------------------------------------------------------------------------------------------------------------------------------------------------------   353
aa37_C12     T------------CCCCTAACCAATGAAAATGG--ACCTTG---------------------------ACCCAACACCCAAAACGGAAAAAATTTTCTTT--------TCAGGTT-----------------------------------   502
aa19_B06     TC----------CCCCAAATC-CTGGAAAATGG--TCCTGA---------------------------ACCAACCTCCCCTAATGGAAAAAGTTTTCTTT--------CCGGGTT-----------------------------------   502
aa33_C08     TC-----------CACAAATCACTGCAAAATGA--TCCTGA---------------------------ACAAACCTCCCAGAATGGAAAAAGTTTTCTTT--------CCTGCTT-----------------------------------   502
NC_AA_1_A10  ------------------------------------------------------------------------------------------------------------------------------------------------------   354
aa28_C03     C------------CCCCCAACACGGGGAAAAGG--ACTTGG---------------------------CCCAAACTCCCCAAAGGGAAAAAAGTTTCTTT--------TCGGGTT-----------------------------------   502
aa29_C04     C------------CCCCCAACACGGGGAAAAGG--ACTTGG---------------------------CCCAAACTCCCCAAAGGGAAAAAAGTTTCTTT--------TCGGGTT-----------------------------------   503
aa30_C05     T------------CCCCAAACCCCGGAAAAAGG--ATTTGG---------------------------CCCAAACCCCCCAAAGGGAAAAAATTTCCTTT--------TCGGGTT-----------------------------------   501
aa43_D06     T------------CCCCAAATCATGGCAAAATG--ATCTTA---------------------------ACCAAACGTCCAAAACGGTACAATATTTCTTT--------TAAGGTT-----------------------------------   502
NC_AA_5_E10  ------------------------------------------------------------------------------------------------------------------------------------------------------   354
aa57_E08     T------------CCCCACATCACGGCATAAAG--ATCATG---------------------------ACCAAAACTCCCAAAAGATAAAAAGTTTCCTT--------TTAAGCT-----------------------------------   502
NC_AA_2_B10  ------------------------------------------------------------------------------------------------------------------------------------------------------   366
aa40_D03     T------------CCTCAAATCACTGCAAAATG--ATCTTG---------------------------ACCAAACATCCCATACTGTAAAAAGTTTCATT--------TCAGGCT-----------------------------------   502
aa44_D07     T------------CCTCAGATCACTGCAAAATG--ATCTTG---------------------------ACCAGACATCCCATACTGTAAAAAGTTTCATT--------TCAGGCT-----------------------------------   497
aa47_D10     T------------CCTCAAATCACTGCAAAATG--ATCTTG---------------------------ACCAAACATCCCATACTGTAAAAAGTTTCATT--------TCAGGCT-----------------------------------   501
aa20_B07     T------------CCTCATATCACTGGAAAATG--ATCTTG---------------------------ACCACACATCCCACACTGGAGAAAGTTTCATT--------TCTTGCT-----------------------------------   498
aa46_D09     T------------CCTCAAATCAATGGAAAATG--AACTTG---------------------------ACCAAACATCCCATACTGTAAAAAGTTTCATT--------TCCGGCT-----------------------------------   502
aa14_B01     T------------CCTCCAAACACTGCAAAATG--AACTTG---------------------------ACCAGACCTCCCATACCGGAAAAAGTTTCATT--------TCAGGGT-----------------------------------   500
aa35_C10     A------------CATCAAACCAAAGTGAACTG--ACCTTG---------------------------ACGACCCATCCCGAATTGAATTCATTTTCATT--------CCTTGAA-----------------------------------   506
aa36_C11     T------------CATCACAGCACAGCAAACTG--AACTTG---------------------------ACCTCACATCCCATACTGAATTTCGTTTCTTT--------TCTTTTT-----------------------------------   503
aa58_E09     C------------CCTCACATCAGTGCAAAGTG--ATCTGG---------------------------ACCACACATCCCATACTGAAAAAAGTTTCTTT--------CCGTGTT-----------------------------------   498
aa51_E02     T------------TCTCAAATCACTGGGAAATG--ATCTTG---------------------------ACCAGACATCCCATACTGAAAAAAGGTTCTTT--------TCATGCT-----------------------------------   501
aa9_A08      T------------CCTATCATCTCTGCACAAAG---CACTGG--------------------------AATAAAATTCCCATAACACACAAAATATAGAA--------TAATGCT-----------------------------------   491
aa39_D02     T------------CCTCTTACCCCTGAAAAATG--ATCATG---------------------------ACCATACACCCCATACCGCAAAAGGTTTAATT--------TTCCGTT-----------------------------------   495
aa41_D04     T------------CCCCATAACTCTGAAAACCG--GTCTTG---------------------------ACCGCACACCCCCCACGGAAAAAAGTTTCATT--------TTGGGGT-----------------------------------   501
aa15_B02     T------------CCTCAAACCGCAGCATGATG--GTGCAC---------------------------ACAACCCCCCCGATGGTGAAAAATGTTTCTTT-----------TGTT-----------------------------------   498
aa52_E03     T------------CATATCATCGCTGAAAGATG--ATGACG---------------------------ACAAAACCTCCACTGTAGAAAATTTTTTTTTT-----------TGCT-----------------------------------   499
aa7_A06      T------------CACAGAATTGCGTGATGATG--ATGACC---------------------------ACACATCCCCCACAGAGGAAAGATTCTTTTTT-----------GGGT-----------------------------------   499
aa23_B10     T------------CCAATTTCCGCAGAAGAATG--TTAAAA---------------------------AAACTTCACCCAAGATAGAAAAATGTTTTAAT-----------TTTT-----------------------------------   499
aa50_E01     C------------CATATAACTGCAGAAAAATG--TTGTCG---------------------------ACCCATCCCCCAAGATCGAAAATTGTTTTTTT-----------TGCT-----------------------------------   499
             .......460.......470.......480.......490.......500.......510.......520.......530.......540.......550.......560.......570.......580.......590.......600
```

CLUSTAL 2.1 MULTIPLE SEQUENCE ALIGNMENT

File: /Users/isabellaloughland/Google Drive/FrankSeebacher3/AA_alignment_3.ps   Date: Mon Feb 26 15:48:34 2018


```
aa12_A11    ------------------------------------------------------------------------------------------------------------------------------------------------------   501
aa21_B08    ------------------------------------------------------------------------------------------------------------------------------------------------------   502
aa24_B11    ------------------------------------------------------------------------------------------------------------------------------------------------------   502
aa11_A10    ------------------------------------------------------------------------------------------------------------------------------------------------------   503
aa25_B12    ------------------------------------------------------------------------------------------------------------------------------------------------------   501
aa13_A12    ------------------------------------------------------------------------------------------------------------------------------------------------------   500
aa38_D01    ------------------------------------------------------------------------------------------------------------------------------------------------------   502
aa45_D08    ------------------------------------------------------------------------------------------------------------------------------------------------------   501
aa48_D11    ------------------------------------------------------------------------------------------------------------------------------------------------------   502
aa31_C06    ------------------------------------------------------------------------------------------------------------------------------------------------------   503
aa49_D12    ------------------------------------------------------------------------------------------------------------------------------------------------------   502
aa10_A09    ------------------------------------------------------------------------------------------------------------------------------------------------------   501
aa18_B05    ------------------------------------------------------------------------------------------------------------------------------------------------------   502
aa22_B09    ------------------------------------------------------------------------------------------------------------------------------------------------------   501
aa8_A07     ------------------------------------------------------------------------------------------------------------------------------------------------------   501
aa17_B04    ------------------------------------------------------------------------------------------------------------------------------------------------------   502
aa56_E07    ------------------------------------------------------------------------------------------------------------------------------------------------------   502
aa54_E05    ------------------------------------------------------------------------------------------------------------------------------------------------------   502
aa16_B03    ------------------------------------------------------------------------------------------------------------------------------------------------------   502
aa53_E04    ------------------------------------------------------------------------------------------------------------------------------------------------------   501
aa32_C07    ------------------------------------------------------------------------------------------------------------------------------------------------------   502
aa26_C01    ------------------------------------------------------------------------------------------------------------------------------------------------------   502
aa34_C09    ------------------------------------------------------------------------------------------------------------------------------------------------------   502
aa6_A05     ------------------------------------------------------------------------------------------------------------------------------------------------------   501
ncaa2_A02   ------------------------------------------------------------------------------------------------------------------------------------------------------   355
aa55_E06    ------------------------------------------------------------------------------------------------------------------------------------------------------   503
aa42_D05    ------------------------------------------------------------------------------------------------------------------------------------------------------   503
NC_AA_4_D10 ------------------------------------------------------------------------------------------------------------------------------------------------------   353
aa37_C12    ------------------------------------------------------------------------------------------------------------------------------------------------------   502
aa19_B06    ------------------------------------------------------------------------------------------------------------------------------------------------------   502
aa33_C08    ------------------------------------------------------------------------------------------------------------------------------------------------------   502
NC_AA_1_A10 ------------------------------------------------------------------------------------------------------------------------------------------------------   354
aa28_C03    ------------------------------------------------------------------------------------------------------------------------------------------------------   502
aa29_C04    ------------------------------------------------------------------------------------------------------------------------------------------------------   503
aa30_C05    ------------------------------------------------------------------------------------------------------------------------------------------------------   501
aa43_D06    ------------------------------------------------------------------------------------------------------------------------------------------------------   502
NC_AA_5_E10 ------------------------------------------------------------------------------------------------------------------------------------------------------   354
aa57_E08    ------------------------------------------------------------------------------------------------------------------------------------------------------   502
NC_AA_2_B10 ------------------------------------------------------------------------------------------------------------------------------------------------------   366
aa40_D03    ------------------------------------------------------------------------------------------------------------------------------------------------------   502
aa44_D07    ------------------------------------------------------------------------------------------------------------------------------------------------------   497
aa47_D10    ------------------------------------------------------------------------------------------------------------------------------------------------------   501
aa20_B07    ------------------------------------------------------------------------------------------------------------------------------------------------------   498
aa46_D09    ------------------------------------------------------------------------------------------------------------------------------------------------------   502
aa14_B01    ------------------------------------------------------------------------------------------------------------------------------------------------------   500
aa35_C10    ------------------------------------------------------------------------------------------------------------------------------------------------------   506
aa36_C11    ------------------------------------------------------------------------------------------------------------------------------------------------------   503
aa58_E09    ------------------------------------------------------------------------------------------------------------------------------------------------------   498
aa51_E02    ------------------------------------------------------------------------------------------------------------------------------------------------------   501
aa9_A08     ------------------------------------------------------------------------------------------------------------------------------------------------------   491
aa39_D02    ------------------------------------------------------------------------------------------------------------------------------------------------------   495
aa41_D04    ------------------------------------------------------------------------------------------------------------------------------------------------------   501
aa15_B02    ------------------------------------------------------------------------------------------------------------------------------------------------------   498
aa52_E03    ------------------------------------------------------------------------------------------------------------------------------------------------------   499
aa7_A06     ------------------------------------------------------------------------------------------------------------------------------------------------------   499
aa23_B10    ------------------------------------------------------------------------------------------------------------------------------------------------------   499
aa50_E01    ------------------------------------------------------------------------------------------------------------------------------------------------------   499
            .......610.......620.......630.......640.......650.......660.......670.......680.......690.......700.......710.......720.......730.......740.......750
```

CLUSTAL 2.1 MULTIPLE SEQUENCE ALIGNMENT

File: /Users/isabellaloughland/Google Drive/FrankSeebacher3/AA_alignment_3.ps   Date: Mon Feb 26 15:48:34 2018


```
aa12_A11     ---------------------------------------------------------------------------AAAAAAAAAAAATTCTTTTT-------------------------------------------------------   521
aa21_B08     ---------------------------------------------------------------------------TTAAAAAAAAAATTCTTTTT-------------------------------------------------------   522
aa24_B11     ---------------------------------------------------------------------------TTAAAAAAAAAAATCCTTTT-------------------------------------------------------   522
aa11_A10     ---------------------------------------------------------------------------TTAAAAAAAAAAATCTTTTT-------------------------------------------------------   523
aa25_B12     ---------------------------------------------------------------------------TTAAAAATAAAATTCTTTTT-------------------------------------------------------   521
aa13_A12     ---------------------------------------------------------------------------TTTAAAATAAAATTCTTTTT-------------------------------------------------------   520
aa38_D01     ---------------------------------------------------------------------------TTTAAAATAAAAATCCTTTA-------------------------------------------------------   522
aa45_D08     ---------------------------------------------------------------------------TTAAAAATAAAAATCCTTTT-------------------------------------------------------   521
aa48_D11     ---------------------------------------------------------------------------TTTAAAATAAAAATCCTTTT-------------------------------------------------------   522
aa31_C06     ---------------------------------------------------------------------------TTTAAAATAAAAATCCTTTT-------------------------------------------------------   523
aa49_D12     ---------------------------------------------------------------------------TTTAAAATAAAAATCCTTTT-------------------------------------------------------   522
aa10_A09     ---------------------------------------------------------------------------TTAAAAAAAAAAACCCTTTT-------------------------------------------------------   521
aa18_B05     ---------------------------------------------------------------------------TTAAAAATAAAATTCTTTTT-------------------------------------------------------   522
aa22_B09     ---------------------------------------------------------------------------TTAAAAAAAAAATTCTTTTT-------------------------------------------------------   521
aa8_A07      ---------------------------------------------------------------------------TTAAAAAAAAAATTCTTTTA-------------------------------------------------------   521
aa17_B04     ---------------------------------------------------------------------------TTAAAAATAAAATTCTTTTA-------------------------------------------------------   522
aa56_E07     ---------------------------------------------------------------------------TTAAAAAAAAAATCCTTTTT-------------------------------------------------------   522
aa54_E05     ---------------------------------------------------------------------------TTAAAAAAAAAATCCTTTTA-------------------------------------------------------   522
aa16_B03     ---------------------------------------------------------------------------TTAAAAAAAAAATCCCTTTT-------------------------------------------------------   522
aa53_E04     ---------------------------------------------------------------------------TTAAAAAAAAAATTCTTTTA-------------------------------------------------------   521
aa32_C07     ---------------------------------------------------------------------------TTAAAAAAAAAATCCTTTTA-------------------------------------------------------   522
aa26_C01     ---------------------------------------------------------------------------TTAAAAAAAAAATTCCTTTA-------------------------------------------------------   522
aa34_C09     ---------------------------------------------------------------------------TTAAAAAAAAAATCCTTTTA-------------------------------------------------------   522
aa6_A05      ---------------------------------------------------------------------------TTAAAAAAAAAATTCTTTTT-------------------------------------------------------   521
ncaa2_A02    ------------------------------------------------------------------------------------------------------------------------------------------------------   355
aa55_E06     ---------------------------------------------------------------------------TTAAAAATAAAAATCTTTTA-------------------------------------------------------   523
aa42_D05     ---------------------------------------------------------------------------TTTAAAAAAAAATCCTTTTT-------------------------------------------------------   523
NC_AA_4_D10  ------------------------------------------------------------------------------------------------------------------------------------------------------   353
aa37_C12     ---------------------------------------------------------------------------TTAAAAAAAAAATTCTTTTT-------------------------------------------------------   522
aa19_B06     ---------------------------------------------------------------------------TTAAAAAAAAAATTCTTTTA-------------------------------------------------------   522
aa33_C08     ---------------------------------------------------------------------------TTAAAAAAAAAATTCTTTTA-------------------------------------------------------   522
NC_AA_1_A10  ------------------------------------------------------------------------------------------------------------------------------------------------------   354
aa28_C03     ---------------------------------------------------------------------------TTAAAAAAAAAATTCCTTTT-------------------------------------------------------   522
aa29_C04     ---------------------------------------------------------------------------TTAAAAAAAAAATTCCTTTT-------------------------------------------------------   523
aa30_C05     ---------------------------------------------------------------------------TTAAAAAAAAAATTCTTTTT-------------------------------------------------------   521
aa43_D06     ---------------------------------------------------------------------------TTAAAAATAAAATTCCTTTT-------------------------------------------------------   522
NC_AA_5_E10  ------------------------------------------------------------------------------------------------------------------------------------------------------   354
aa57_E08     ---------------------------------------------------------------------------TTTAAAAAAAAAATCCTTTT-------------------------------------------------------   522
NC_AA_2_B10  ------------------------------------------------------------------------------------------------------------------------------------------------------   366
aa40_D03     ---------------------------------------------------------------------------TTTAAAATAAAAATCCTTTT-------------------------------------------------------   522
aa44_D07     ---------------------------------------------------------------------------TTTAAAATAAAAATCCTTTT-------------------------------------------------------   517
aa47_D10     ---------------------------------------------------------------------------TTTAAAATAAAAATCCTTTT-------------------------------------------------------   521
aa20_B07     ---------------------------------------------------------------------------TTTAAAATAAAAATCCTTTT-------------------------------------------------------   518
aa46_D09     ---------------------------------------------------------------------------TTTAAAATAAAAATCCTTTT-------------------------------------------------------   522
aa14_B01     ---------------------------------------------------------------------------TTTAAAATAAAAATCCTTTT-------------------------------------------------------   520
aa35_C10     ---------------------------------------------------------------------------TTTAAAATTAATTTCCTTTA-------------------------------------------------------   526
aa36_C11     ---------------------------------------------------------------------------TATAAAATAAATTTCCTTTT-------------------------------------------------------   523
aa58_E09     ---------------------------------------------------------------------------TTTAAAAAAAAAATCATTTT-------------------------------------------------------   518
aa51_E02     ---------------------------------------------------------------------------TTTAAAATAAAAATCCTTTT-------------------------------------------------------   521
aa9_A08      ---------------------------------------------------------------------------TTTATACTAAAAATAAAATT-------------------------------------------------------   511
aa39_D02     ---------------------------------------------------------------------------TTTGGATTTAAAATCAATTT-------------------------------------------------------   515
aa41_D04     ---------------------------------------------------------------------------TTTAAAAAAAAAAACCTTTT-------------------------------------------------------   521
aa15_B02     ---------------------------------------------------------------------------GTTAAAATAAAAATCCTTTT-------------------------------------------------------   518
aa52_E03     ---------------------------------------------------------------------------GTTTTTATAAAAATCTTTTT-------------------------------------------------------   519
aa7_A06      ---------------------------------------------------------------------------TTTATAAAAAAAATCTTTTT-------------------------------------------------------   519
aa23_B10     ---------------------------------------------------------------------------TTTTTAAGAATAAAAATTTT-------------------------------------------------------   519
aa50_E01     ---------------------------------------------------------------------------TTTTTAAAAAAAAACCTTTT-------------------------------------------------------   519
             .......760.......770.......780.......790.......800.......810.......820.......830.......840.......850.......860.......870.......880.......890.......900
```

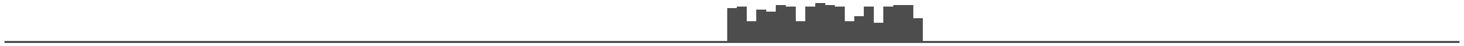

CLUSTAL 2.1 MULTIPLE SEQUENCE ALIGNMENT


```
aa12_A11      ----------------------------------------------------------ATTCAGGAATGATT----------------------------------ATTACGAACAACCC------------------------------   549
aa21_B08      ----------------------------------------------------------CTTCGATAATGATA----------------------------------ATAAGAAACACCCC------------------------------   550
aa24_B11      ----------------------------------------------------------ATTCATCAATGATT----------------------------------AAAAGAAAAAAAAC------------------------------   550
aa11_A10      -----------------------------------------------------------ATTCAGCATGGATT---------------------------------ATAAGAACCAATAA------------------------------   551
aa25_B12      -----------------------------------------------------------ATTCACCAAGGATA---------------------------------ATAAGAACCAATAA------------------------------   549
aa13_A12      -----------------------------------------------------------ATTCACCAAGGATA---------------------------------ATAATAACCAATAA------------------------------   548
aa38_D01      -----------------------------------------------------------ATTCACCAAGGATT---------------------------------ATAATGACCAATAA------------------------------   550
aa45_D08      -----------------------------------------------------------ATTCACCAATGATT---------------------------------ATAATGACCAATAA------------------------------   549
aa48_D11      -----------------------------------------------------------ATTCACCATTGATT---------------------------------ATTATGACCAATAA------------------------------   550
aa31_C06      -----------------------------------------------------------ATTCACCAATGATA---------------------------------ATAATGACCAATAA------------------------------   551
aa49_D12      -----------------------------------------------------------ATTCAACATTGATA---------------------------------ATAATAACCAAAAC------------------------------   550
aa10_A09      -----------------------------------------------------------ATTCACCATGGATA---------------------------------ATAAGAACAAAAAA------------------------------   549
aa18_B05      -----------------------------------------------------------ATTCGACAATGATA---------------------------------ATAAGAACCAATAA------------------------------   550
aa22_B09      -----------------------------------------------------------ATTCACCATGGATT---------------------------------ATAAGAACCAAAAC------------------------------   549
aa8_A07       -----------------------------------------------------------ATTCACAATGGATA---------------------------------ATAAGAACCAAAAC------------------------------   549
aa17_B04      -----------------------------------------------------------ATTCACCATGGATA---------------------------------TTATGAACAATTAC------------------------------   550
aa56_E07      -----------------------------------------------------------ATTCACCATGATTA---------------------------------ATAAGAACCAATAA------------------------------   550
aa54_E05      -----------------------------------------------------------ATTCACCATGGATA---------------------------------ATATGAACCAATAA------------------------------   550
aa16_B03      -----------------------------------------------------------ATTCACCATGGATA---------------------------------ATATGAACCAAAAA------------------------------   550
aa53_E04      -----------------------------------------------------------ATTAACCATGAATA---------------------------------TTATGAGCAATAAA------------------------------   549
aa32_C07      -----------------------------------------------------------ATTAACAATGAATA---------------------------------ATAAGAACAATAAC------------------------------   550
aa26_C01      -----------------------------------------------------------ATTCACAATGAATA---------------------------------ATAAGAACCATAAC------------------------------   550
aa34_C09      -----------------------------------------------------------ATTCACAATGAATA---------------------------------ATAAGAACAAAAAC------------------------------   550
aa6_A05       -----------------------------------------------------------ATTCACCATGAATA---------------------------------ATAAGAACAATAAC------------------------------   549
ncaa2_A02     ------------------------------------------------------------------------------------------------------------------------------------------------------   355
aa55_E06      -----------------------------------------------------------ATTTT-TTTGATTA---------------------------------ATATGAACCATAAC------------------------------   550
aa42_D05      -----------------------------------------------------------ATTCGCCATGATTA---------------------------------TTAAGAACCAAAAA------------------------------   551
NC_AA_4_D10   ------------------------------------------------------------------------------------------------------------------------------------------------------   353
aa37_C12      -----------------------------------------------------------ATTCATCATTAATA---------------------------------ATAAGAACCAAAAC------------------------------   550
aa19_B06      -----------------------------------------------------------ATTAACAATGATTA---------------------------------TTAAGAACAAAAAC------------------------------   550
aa33_C08      -----------------------------------------------------------TTTAACAATGATTA---------------------------------ATAAGAACAACAAC------------------------------   550
NC_AA_1_A10   ------------------------------------------------------------------------------------------------------------------------------------------------------   354
aa28_C03      ----------------------------------------------------------TTTCCACAAGGATT---------------------------------AATAAGACCCAAAA-------------------------------   550
aa29_C04      ----------------------------------------------------------TTTCCACAAGGATT---------------------------------AATAAGACCCAAAA-------------------------------   551
aa30_C05      ----------------------------------------------------------TTTCACCAAGAATT----------------------------------ATTAGAAACAAAAC------------------------------   549
aa43_D06      -----------------------------------------------------------ATTAACCATGAATA---------------------------------ATAACAACCAAAAA------------------------------   550
NC_AA_5_E10   ------------------------------------------------------------------------------------------------------------------------------------------------------   354
aa57_E08      -----------------------------------------------------------ATTAAAAAAAGAAT--------------------------------ATTAAGAACAATAA-------------------------------   550
NC_AA_2_B10   ------------------------------------------------------------------------------------------------------------------------------------------------------   366
aa40_D03      ----------------------------------------------------------ATTCAGCAATGATT---------------------------------ATTATGAGCAATAA-------------------------------   550
aa44_D07      ----------------------------------------------------------ATTCACCAATGATT---------------------------------ATTATGAGCAATAA-------------------------------   545
aa47_D10      ----------------------------------------------------------ATTTAGCAATGATT---------------------------------ATTATGAGCAATAA-------------------------------   549
aa20_B07      ----------------------------------------------------------ATTTATCAATGATT---------------------------------AATAAGAACAATAA-------------------------------   546
aa46_D09      ----------------------------------------------------------ATTTTTCTAAGAAT---------------------------------ATTATGAGCAAAAA-------------------------------   550
aa14_B01      ----------------------------------------------------------ATTTAACAATGAAT---------------------------------AATAAGAACAAAAA-------------------------------   548
aa35_C10      ----------------------------------------------------------ATATGGAAAAGAAT--------------------------------AAACAAACCCCAAC--------------------------------   554
aa36_C11      ----------------------------------------------------------ATTCAGCATAGAAT-------------------------------AAAATGAACAACAC---------------------------------   551
aa58_E09      ----------------------------------------------------------TTTTTGCTGAGAAT---------------------------------AATAAGAAAAAAAC-------------------------------   546
aa51_E02      ----------------------------------------------------------ATTTAGAAAAGAAT----------------------------------ATAAAGAACAAAAC------------------------------   549
aa9_A08       ----------------------------------------------------------AATTATTTTTTATT---------------------------------ACAAAGAAGAATAA-------------------------------   539
aa39_D02      ----------------------------------------------------------ACTTTTCATTGATT---------------------------------ATTATGAACAATAA-------------------------------   543
aa41_D04      ----------------------------------------------------------ATACTTCAATGTTT---------------------------------TGTATTAAAAATAA-------------------------------   549
aa15_B02      -----------------------------------------------------------TTTTTACAACGATT--------------------------------AATAATAAAAAAAA-------------------------------   546
aa52_E03      -----------------------------------------------------------TTTTTTCAGCGATG---------------------------------ATTATGAGCAGCAA------------------------------   547
aa7_A06       -----------------------------------------------------------TTTTATCAATGATA---------------------------------ATTATAAGCAACAA------------------------------   547
aa23_B10      -----------------------------------------------------------TTAATCCTTTAATG---------------------------------ATAATAAGAAAAAA------------------------------   547
aa50_E01      -----------------------------------------------------------TTTCCCCACTGATA---------------------------------ATTATGAGAAATAA------------------------------   547
              .......910.......920.......930.......940.......950.......960.......970.......980.......990......1000......1010......1020......1030......1040......1050
```

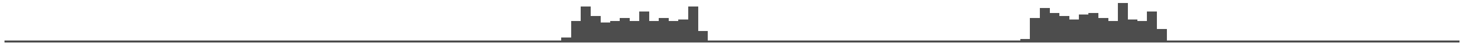

CLUSTAL 2.1 MULTIPLE SEQUENCE ALIGNMENT

File: /Users/isabellaloughland/Google Drive/FrankSeebacher3/AA_alignment_3.ps   Date: Mon Feb 26 15:48:34 2018


```
aa12_A11     ------------CCCACAGAATGTAGTCTGGTTTGATTTG------TTTGAAAAAAAAA----------------------------------------------------------------------TATTTTTATT-----------   600
aa21_B08     ------------CCCTGT-AATGGGGTTTGATTTGATTTT------GTTGGAAAAAAAA----------------------------------------------------------------------AAATTTTATT-----------   600
aa24_B11     ------------CCCCCCAAAAGTCTTGGGGTTTTATTTT------GTTGGTTAAAAAA----------------------------------------------------------------------AATTTTAATT-----------   601
aa11_A10     ------------CCCTCATATGGAATTCGGGTTTGATTTG------TTTGTCGGAAAAA-----------------------------------------------------------------------AATTCCAATT----------   602
aa25_B12     ------------CCCTCTAAAGGAATTCGGGTTGGATTGG------TTTGTCGGAAAAA-----------------------------------------------------------------------AATTCCATTT----------   600
aa13_A12     ------------CCCTCTTATTGAATTCGGTTTTGATTGG------TTTGTCTAAAAAA-----------------------------------------------------------------------AATTTCAATT----------   599
aa38_D01     ------------CCCTCATAATGTATTCGGTTTTGATTTG------TTTGTCTGAAAAA-----------------------------------------------------------------------GATTCCAATT----------   601
aa45_D08     ------------CCCTCATATGGTATTCGGTTTGGATTTG------TTTGTCTGAAAAA-----------------------------------------------------------------------AATTCCAATT----------   600
aa48_D11     ------------CCCTCAAATTGTATCCGGTTTGGATTGG------TTGGTCTGAAAAA-----------------------------------------------------------------------GATTCCATTT----------   601
aa31_C06     ------------CCCTCAAAAGGTATCCGGTTTGGATTTG------TTTGTCTGAAAAA-----------------------------------------------------------------------AATTCCAATT----------   602
aa49_D12     ------------CCCTCAAAAGGTATTCGGGTTTGATTTG------TTTGCCTGAAAAA-----------------------------------------------------------------------AATTTCATTT----------   601
aa10_A09     ------------CCCCAATAAGGTATCCGGGTTTGATTTG------TTGGTCGAAAAAA-----------------------------------------------------------------------AATTTCAATT----------   600
aa18_B05     ------------CCCTCAAATGGTATCCGGTTTGGATTTG------TTGGCCAGAAGAA-----------------------------------------------------------------------AATTTCATTT----------   601
aa22_B09     ------------CCCTCAAATTGAATTCGGTTTTGTTTGG------TTGGTCGAAAAAA-----------------------------------------------------------------------AATTTCATTT----------   600
aa8_A07      ------------CCCCCATATGGAATTCGGTTTTATTTGG------TTGGCCGAAAAAA-----------------------------------------------------------------------AATTTCATTT----------   600
aa17_B04     ------------CCTTCTTATGGAATTCGGTTTTGATTTG------TTTGTCTGAAGAG-----------------------------------------------------------------------AATTTCATTT----------   601
aa56_E07     ------------CCTTCATATTGTATCCGGTTTTGATTTG------TTTGTTGAAAGAA-----------------------------------------------------------------------AATTTCATTT----------   601
aa54_E05     ------------CCTCATAATGGTATTCGGTTTGAATTGG------TTTGTCTAAAAAA-----------------------------------------------------------------------AATTTCATTT----------   601
aa16_B03     ------------CCCCATAATGGTATTCGGTTTGGATTGG------TTTGTCTAAAGAA-----------------------------------------------------------------------AATTCAATTT----------   601
aa53_E04     ------------CCCTCTTATG-TATTCGGTTTGATTTGT-------TTGTCTGAAAGAA----------------------------------------------------------------------AATTCCATTT----------   599
aa32_C07     ------------CCTCATTATGGAATTCGGTTTGATTTGG-------TTGGCTGAAAGAA----------------------------------------------------------------------AATTTCAATT----------   601
aa26_C01     ------------CCCCAAAAAGGATTCCGGTTTGATTTGT-------TTGTCCGGAAAAA----------------------------------------------------------------------AATTTCATTT----------   601
aa34_C09     ------------CCCTAAAAAGTATTCCGGTTTGAATTGT-------TTTGCCG-AAAAA----------------------------------------------------------------------AAATTCAATT----------   600
aa6_A05      ------------CCCCCAAAAGGAATTCGGTATTGATTTG------TTTGACTGAAAAA-----------------------------------------------------------------------AATTTCATTT----------   600
ncaa2_A02    ------------------------------------------------------------------------------------------------------------------------------------------------------   355
aa55_E06     ------------CCCCAAAAAGGAATCCGGATTCAATTTT------TTGTTTTGAAAAA-----------------------------------------------------------------------AATTCAAATT----------   601
aa42_D05     ------------CCCCCAAATGGATTCCGGTTTTAATTTT------TTTGCCT-AAAAA-----------------------------------------------------------------------AATTCAAATT----------   601
NC_AA_4_D10  ------------------------------------------------------------------------------------------------------------------------------------------------------   353
aa37_C12     ------------CCCCCATACGGTATTCGGTTTTGAGTTG------TTTGCAGGAAAAA-----------------------------------------------------------------------AATTTCATTT----------   601
aa19_B06     ------------CCCCCAAATGTAATCCGGTTTGATTTGG------TTGGAAGAAAAAA------------------------------------------------------------------------ATTTCTATTT---------   601
aa33_C08     ------------CCCCAGAATGTAGTCTGGTTTGATTTGT------TTGGCTGAAAAAT------------------------------------------------------------------------ATTTTTATTT---------   601
NC_AA_1_A10  ------------------------------------------------------------------------------------------------------------------------------------------------------   354
aa28_C03     -----------CGCCCTAAAGT-TTTCCGGGTTGGTTTGA------TTTGCCTGAAAAA------------------------------------------------------------------------ATTTTTTTTT---------   601
aa29_C04     -----------CGCCCTAAAGT-TTTCCGGGTTGGTTTGA------TTTGCCTGAAAAA------------------------------------------------------------------------ATTTTTTTTT---------   602
aa30_C05     ------------CCCCCAAAGTGTTTTCGGTTTAAATTTG------TTTGCCAAAAAAA------------------------------------------------------------------------ATTTCAATTT---------   600
aa43_D06     ------------CCCTCCTAAGGTATCCGGTTTTGATTTG------TTGGACTGAAAAA-----------------------------------------------------------------------TATTTCTTTT----------   601
NC_AA_5_E10  ------------------------------------------------------------------------------------------------------------------------------------------------------   354
aa57_E08     ------------CCCTCAAAAAGTATGAGCGGTTTATCTG------TTTGTGAGATCGA-----------------------------------------------------------------------TATTTTTTTT----------   601
NC_AA_2_B10  ------------------------------------------------------------------------------------------------------------------------------------------------------   366
aa40_D03     ------------CCCTCATAATGTATTCGGGTTTGATTTG------TTTGTCTGAAAAA-----------------------------------------------------------------------AATTTCAATT----------   601
aa44_D07     ------------CCCTCATAATGTATTCGGGTTTGATTTG------TTTGTCTGAAAGA-----------------------------------------------------------------------GATTTCAATT----------   596
aa47_D10     ------------CCCTCATAATGTATTCGGGTTTGATTTG------TTTGTCTGAAAAA-----------------------------------------------------------------------AATTTCAATT----------   600
aa20_B07     ------------CCCTCCTAACGGATTCGGGTTTGATTTG------TTTGTCTGAAAGA-----------------------------------------------------------------------AAATTCAATT----------   597
aa46_D09     ------------CCCTCCCAATGGATTCGGGTTTGATTTG------TTTGTCTGAAAGA-----------------------------------------------------------------------AAATTCAATT----------   601
aa14_B01     ------------CCCTCCTAATGGAATTCGGTTTGATTTG------GTTGTCTGAAAAA-----------------------------------------------------------------------AAATTCAATT----------   599
aa35_C10     ------------CCATCGTATTGGGTTTGGATTTGATTGG------TTGAACTAAAT----------------------------------------------------------------------------TTTATTT----------   600
aa36_C11     ------------CCCTCATATTGTATTCTGATTTGATTTG------TTTGACTAAAA----------------------------------------------------------------------------TATATTT----------   597
aa58_E09     ------------CCCACCCAGTGTATTCGG-TTTGGTTTGT-----TTTGTCTAAAAGA-----------------------------------------------------------------------AATTTAATTT----------   597
aa51_E02     ------------CCCTCATAATGTATTCGGTTTTGATTTT------GTTGTTTAAAATA-----------------------------------------------------------------------AATTTTTTTT----------   600
aa9_A08      ------------TACTCACAACGTATTCCGGTTTGATTTG------TTTTTTTAAT----------------------------------------------------------------------TTATAGTTTAAAT-----------   590
aa39_D02     ------------CCCTCACCCTGCATACGGGTTTGAGTTT------TTTGTCTGAG----------------------------------------------------------------------GCAGAAATAAATT-----------   594
aa41_D04     ------------CCCCCAAATTGTATTCGGGTTTGATTTG------TTTGTCTGAAAGA----------------------------------------------------------------------GATTTCAGTT-----------   600
aa15_B02     ------------CACCCCCAGTATTGTCGTTTTGGTTGTT------TGTGTGATAGAAAT---------------------------------------------------------------------AAATTTTTTT-----------   598
aa52_E03     ------------CCCCCCTCATGTTGTCGGGTGGGATGAT------TTTGTCGGAGAAAA---------------------------------------------------------------------AATATTTTTT-----------   599
aa7_A06      ------------CCCCCCCAAAGTGGTCTCCTTTGTTTTG------TTTGTGTGATAAAA---------------------------------------------------------------------ATATCTTTTT-----------   599
aa23_B10     -----------CCACCCACAAACC--TCATTCTGGAATTAGTGG--TTGGT-AGGAAGAC---------------------------------------------------------------------TGAATTAAAT-----------   601
aa50_E01     ------------CCCCCAAAAAGTA-TCAT-CGGGTTTTGTTTT--TTTGTGAGAAAGAT---------------------------------------------------------------------TAATTTAAAT-----------   601
             ......1060......1070......1080......1090......1100......1110......1120......1130......1140......1150......1160......1170......1180......1190......1200
```

CLUSTAL 2.1 MULTIPLE SEQUENCE ALIGNMENT

File: /Users/isabellaloughland/Google Drive/FrankSeebacher3/AA_alignment_3.ps   Date: Mon Feb 26 15:48:34 2018


```
aa12_A11     ----------------------------------TTTTTTTTTTAATA----------------------------------------AAAAAAAAAAAATTACGCTCAGGAAGGGGGGGGG----------GGCCCAA--AACCAA---   661
aa21_B08     ----------------------------------TTTTTTTTTTTATT----------------------------------------AAAAAAAAAAAAATCTGCTCTGGTATGGGGGGGG-----------GGGAAA--AACCAAA--   661
aa24_B11     ----------------------------------TTTTTTTTTTTTTT-----------------------------------------AAAAAAAAAAAAAAAACTTAGGAGGGGGGGGGGG---------GGGCCAACCAAACAAACC   667
aa11_A10     ----------------------------------TAATTTTTTTATTT----------------------------------------AAATAAATAGAATAAGCCTCTGT---ATGGGTAGGG----------GTTTGGGCCCAA----   661
aa25_B12     ----------------------------------AAATTTTTTAATTT----------------------------------------AAATAAATAAAATAAGGCTCTGT---AAGGTAAGGG----------GTTTTGGGCCAAA---   660
aa13_A12     ----------------------------------TAATTTTTTTATTT----------------------------------------AAATAAATAGAATAAGCTCCTGT---ATGGGTAGGG----------TTTTTGCCCCAAA---   659
aa38_D01     ----------------------------------TAATTTTTTTATTT----------------------------------------AAATAAATAAAATAAGGCTCTGT---AAGGGTAGGG----------GTTTTGGGCCACATA-   663
aa45_D08     ----------------------------------TAATTTTTTAATTT----------------------------------------AAATAAATAAAATTAGGCTCGGT---AAGGGTTGGG-----------TTTTGGGCCACAA--   660
aa48_D11     ----------------------------------AAATTTTTTAATTA----------------------------------------AAAAAAATAAAATTAGGCTCTGT---AAGGGTATGG----------GGTTTGGGCCACA---   661
aa31_C06     ----------------------------------AAATTTTTTTATTT----------------------------------------AATTAAATAAAATAAGGCTCGGT---AAGGTTATGG---------GGTTGGGGCCCACA---   663
aa49_D12     ----------------------------------TAATTTTTTTATTT----------------------------------------AAATAAATAAATTAAGCCTCGGT---ACGGTAATGG----------GATTTGTGCCCCAGCC   664
aa10_A09     ----------------------------------TAATTTTTTTATTT----------------------------------------AAAAAAA-AAAATTAGCTTCTGT---AGGG--ATGG----------GGTTTGGGCCCCAACA   660
aa18_B05     ----------------------------------TATTTTTTTAATTA----------------------------------------AAAAAAATAAGATAAGCTTCGGT---ATGGGTTGGG-----------TTTG--ACCCCAA--   659
aa22_B09     ----------------------------------TATTTTTTTTATTT----------------------------------------AATAAAATAAAATAAGGTTCGGT---ATGGAGTGGG-----------TTTGGAGCCCCAATC   662
aa8_A07      ----------------------------------AAATTTTTTAATTA----------------------------------------AAAAAAATAAAATAAGCCTCGGA---ATGGTAAGGG----------GTTTG--GGCCCA---   658
aa17_B04     ----------------------------------TAGTTTTTTTATTT----------------------------------------AATAAAATAAAATAAGCTTCTGT---ATGGTAGGGG-----------GGTG-TGCCCAAGCC   662
aa56_E07     ----------------------------------TAGTTTTTTATTTT----------------------------------------AAATAAAAAAAATAGGCTTCTGT---ATGGTAGGGG-----------TTTTTTGCCCAA---   660
aa54_E05     ----------------------------------TAGTTTTTTTATTT----------------------------------------AAATAAATAAAATAAGCTCCGTA---ATGGTTTGGT-----------GTGTGTGCCAAAA--   661
aa16_B03     ----------------------------------TAATTTTTTTATTT----------------------------------------AATAAAATAAAATAAGGCTCTTA---ATGGTAAGGG-----------TTTTTGGCCCAA---   660
aa53_E04     ----------------------------------AAG-TTTTTTATTT----------------------------------------AATAAAATAGAATAAGCTCCTGT---ATGGGAAGG------------GTTTGTGCCCAAA--   657
aa32_C07     ----------------------------------TAGTTTTTTAATTA----------------------------------------AATAAAATAAAATAAGCTCCTGA---ATGGAGTGG------------TTTTGGACCAAAA--   660
aa26_C01     ----------------------------------TAATTTTTTAATTT----------------------------------------AATTAAATAAAATAAGCTCCTGA---ATGGTAGGGT-----------GTTTGGGCCCAA---   660
aa34_C09     ---------------------------------TTAGTTTTTTAATT-----------------------------------------AAATAAAAAAAATAAGCTTCGTA---ATGGTAAGGG-----------GTTTGG-CCCA----   657
aa6_A05      ----------------------------------TATTTTTTTTATTT----------------------------------------AATTAAAAAAAATAAGGTTCTGG---ATGGG-AGGG-----------GGGGGGACCCAA---   658
ncaa2_A02    ------------------------------------------------------------------------------------------------------------------------------------------------------   355
aa55_E06     ----------------------------------TAATTTTTTAATTT----------------------------------------AAATAAATAAAATTAGGATTAGG--ATCGGTAGTG------------GTTTTGCCCAAAATG   663
aa42_D05     ----------------------------------TATTTTTTTATTTT----------------------------------------AATAAAATAGAATAAG-CTCCGT----ATGTGGTGTG-----------GGGGGGCCCCAAAA   661
NC_AA_4_D10  ------------------------------------------------------------------------------------------------------------------------------------------------------   353
aa37_C12     ----------------------------------TATTTTTTTTATTT----------------------------------------AATTAAATAGAATTAGGCTCTGT--ATTGGTAGGG------------AGTTAGGCCCAAATC   663
aa19_B06     ----------------------------------TATTTTTTTATTTA----------------------------------------AAAAAAAAAAAATTAGCTTTGG----ATGGGAGGGG----------GGTT-CGACCCAAA--   660
aa33_C08     ----------------------------------TTTTTTTTTAATTA----------------------------------------AATAAAAAAAAATAAGCTCTGGG---ATGGGGGGGG----------GGTTGCAACACAAA--   662
NC_AA_1_A10  ------------------------------------------------------------------------------------------------------------------------------------------------------   354
aa28_C03     ----------------------------------TATTTTTTTTATT-----------------------------------------AAAAAAAAAA-ATTAGCGTTG-T---GTGGTGTGGG----------ATTT--GGCCCAAACA   659
aa29_C04     ----------------------------------TATTTTTTTTATT-----------------------------------------AAAAAAAAAA-ATTAGCGTTG-T---GTGGTGTGGG----------ATTT--GGCCCAAACA   660
aa30_C05     ----------------------------------TATTTTTTTTATT-----------------------------------------AAAAAAAAAAGATTAGCCTCT-G---AAG--GTAGG----------GGTT--GGCCCAAA--   655
aa43_D06     ----------------------------------TATTTTTTTTATTT----------------------------------------AATTTAATACAATTAGGTTCTGT--AGTGGTAGGC---------GGAATGGCCCCACGAAAT   666
NC_AA_5_E10  ------------------------------------------------------------------------------------------------------------------------------------------------------   354
aa57_E08     ----------------------------------TTATTTTTTTATTT----------------------------------------AAAAAAATAAAATTTTGCTCTTT---AGGGTATAGGG---------AATTTGCCCCCAAAAA   665
NC_AA_2_B10  ------------------------------------------------------------------------------------------------------------------------------------------------------   366
aa40_D03     ----------------------------------TAATTTTTTTATTT----------------------------------------AAATAAATAAAATTAGGCTCTGTA--ATGGTATGGG----------GGTTTGGGCCCAACA-   664
aa44_D07     ----------------------------------TAAGTTTTTTATTT----------------------------------------ATATAAATAAAATTAAGCTCTGTA--ATGGTATGGG----------GGTGTG--CCCAAAAA   658
aa47_D10     ----------------------------------TAAGTTTTTTATTT----------------------------------------AAATAAAAAAAATTAGGCTCTGTA--ATGGTATGGG----------GTTTTTGTCCCACAAA   664
aa20_B07     ----------------------------------TAATTTTTTTATTT----------------------------------------AAATAAAAAAAATTAAGCTCTGTA--ATGGGATGGG----------GTTGAG--CCCACAAA   659
aa46_D09     ----------------------------------TAAGTTTTTTATTT----------------------------------------AAATAAAAAAAAATAAGCTCTGTA--ATGGTATGGG----------GTTGGTG-CCCACAAA   664
aa14_B01     ----------------------------------TAAGTTTTTTATTT----------------------------------------TAATAAAATAGAATAAGCTCTGTA--ATGGTATGGG----------GTTGGGG-CCCAAACC   662
aa35_C10     ----------------------------------TATTTTATTTATTT----------------------------------------AATTAAATAAAATAAGGGTAGGTA--GGGGAATGGG------------GCGAGGCCCCAACC   662
aa36_C11     ----------------------------------TAATTTTTTTTTTT----------------------------------------TATTAAAAAAAATTAGACTATGCT--ATGTAGTGGG------------GTGGAGCCCCAACC   659
aa58_E09     ----------------------------------TATTTTTTTTTTTA----------------------------------------AATTAAAAAAAATAAGGCTCTGTA--GTGGTGGGGG-----------GGGGGAGCCCAAACC   660
aa51_E02     ----------------------------------TTTTTTTTTTTTTT----------------------------------------AAAAAAAAAAGAATAGGCTCTGTG--ATGGTATGAT-------------GTGTGCTCCACCC   661
aa9_A08      ----------------------------------TAATTTTTTTTTTT----------------------------------------TATTTTTAAATAAAAGACTTTGTA--CTGGGCTGTG-----------GGGGGGGCCAAGGGC   653
aa39_D02     ----------------------------------TAAATTTTTTATTT----------------------------------------TTATTTAAAAAATAAAGCATTGTG--TTGGTATGGG-----------GTTTTGGCCCCATG-   656
aa41_D04     ----------------------------------TTAATTTTTTATTT----------------------------------------ATATAAATAAAATAAAGCTCTGTG--AAGGTTTGGG-----------GTGTGAGCAACAAAA   663
aa15_B02     ----------------------------------TTTTTTTTTTTTTT-------------------------------------------TAAAAAAAATAATAATATCTGCAGGGGGGGGGG----------GGTAG-CCCACACCAA   660
aa52_E03     ----------------------------------TTTTTTTTTTTTAT-------------------------------------------TAAAAAAAAAATTATTCTGCGCAGTGGTAGGGG----------GGTGGTGGCACAAAAA   662
aa7_A06      ----------------------------------TATTTTTTTTTTT----------------------------------------------AAATAAAAAATATAATGCGCTGTGGGGGGGG----------GGGGTGGTGACCACAA   659
aa23_B10     ----------------------------------TTTTTTTTTTATTA-------------------------------------------TAAAATAAAAAG---ACTGGGAAG-GCTGGGGG----------GGGGTGGCAACAAATG   660
aa50_E01     ----------------------------------TTTTTTTTTTAATT-------------------------------------------TAAAAAAAATAATTCGCTTTGGGG-GGGGGGGG----------GGGGTGCCAACAAGTG   663
             ......1210......1220......1230......1240......1250......1260......1270......1280......1290......1300......1310......1320......1330......1340......1350
```

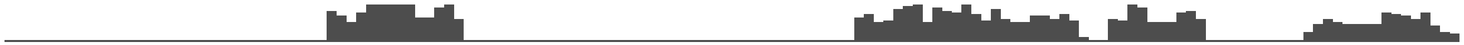

CLUSTAL 2.1 MULTIPLE SEQUENCE ALIGNMENT


```
aa12_A11    ------------------------------------------------------------------------------------------------------------------------------------------------------   661
aa21_B08    ------------------------------------------------------------------------------------------------------------------------------------------------------   661
aa24_B11    AGGATG------------------------------------------------------------------------------------------------------------------------------------------------   673
aa11_A10    ------------------------------------------------------------------------------------------------------------------------------------------------------   661
aa25_B12    ------------------------------------------------------------------------------------------------------------------------------------------------------   660
aa13_A12    ------------------------------------------------------------------------------------------------------------------------------------------------------   659
aa38_D01    ------------------------------------------------------------------------------------------------------------------------------------------------------   663
aa45_D08    ------------------------------------------------------------------------------------------------------------------------------------------------------   660
aa48_D11    ------------------------------------------------------------------------------------------------------------------------------------------------------   661
aa31_C06    ------------------------------------------------------------------------------------------------------------------------------------------------------   663
aa49_D12    CA----------------------------------------------------------------------------------------------------------------------------------------------------   666
aa10_A09    ------------------------------------------------------------------------------------------------------------------------------------------------------   660
aa18_B05    ------------------------------------------------------------------------------------------------------------------------------------------------------   659
aa22_B09    AA----------------------------------------------------------------------------------------------------------------------------------------------------   664
aa8_A07     ------------------------------------------------------------------------------------------------------------------------------------------------------   658
aa17_B04    ACC---------------------------------------------------------------------------------------------------------------------------------------------------   665
aa56_E07    ------------------------------------------------------------------------------------------------------------------------------------------------------   660
aa54_E05    ------------------------------------------------------------------------------------------------------------------------------------------------------   661
aa16_B03    ------------------------------------------------------------------------------------------------------------------------------------------------------   660
aa53_E04    ------------------------------------------------------------------------------------------------------------------------------------------------------   657
aa32_C07    ------------------------------------------------------------------------------------------------------------------------------------------------------   660
aa26_C01    ------------------------------------------------------------------------------------------------------------------------------------------------------   660
aa34_C09    ------------------------------------------------------------------------------------------------------------------------------------------------------   657
aa6_A05     ------------------------------------------------------------------------------------------------------------------------------------------------------   658
ncaa2_A02   ------------------------------------------------------------------------------------------------------------------------------------------------------   355
aa55_E06    CCAAA-------------------------------------------------------------------------------------------------------------------------------------------------   668
aa42_D05    A-----------------------------------------------------------------------------------------------------------------------------------------------------   662
NC_AA_4_D10 ------------------------------------------------------------------------------------------------------------------------------------------------------   353
aa37_C12    ACAA--------------------------------------------------------------------------------------------------------------------------------------------------   667
aa19_B06    ------------------------------------------------------------------------------------------------------------------------------------------------------   660
aa33_C08    ------------------------------------------------------------------------------------------------------------------------------------------------------   662
NC_AA_1_A10 ------------------------------------------------------------------------------------------------------------------------------------------------------   354
aa28_C03    ------------------------------------------------------------------------------------------------------------------------------------------------------   659
aa29_C04    ------------------------------------------------------------------------------------------------------------------------------------------------------   660
aa30_C05    ------------------------------------------------------------------------------------------------------------------------------------------------------   655
aa43_D06    TGAA----------------------------------------------CAGAGCCCA-------------------------------------------------------------------------------------------   679
NC_AA_5_E10 ------------------------------------------------------------------------------------------------------------------------------------------------------   354
aa57_E08    TTGGAT----------------------------------------------GGGGCCAAAGCCCAA-----------------------------------------------------------------------------------   686
NC_AA_2_B10 ------------------------------------------------------------------------------------------------------------------------------------------------------   366
aa40_D03    ------------------------------------------------------------------------------------------------------------------------------------------------------   664
aa44_D07    AAAAAA----------------------------------------------AAAAAAA-------------------------------------------------------------------------------------------   671
aa47_D10    ------------------------------------------------------------------------------------------------------------------------------------------------------   664
aa20_B07    ACACCA----------------------------------------------TCCA----------------------------------------------------------------------------------------------   669
aa46_D09    CAAA--------------------------------------------------------------------------------------------------------------------------------------------------   668
aa14_B01    CA----------------------------------------------------------------------------------------------------------------------------------------------------   664
aa35_C10    CAA---------------------------------------------------------------------------------------------------------------------------------------------------   665
aa36_C11    CCACCCAAA---------------------------------------------------------------------------------------------------------------------------------------------   668
aa58_E09    AAA---------------------------------------------------------------------------------------------------------------------------------------------------   663
aa51_E02    AAAAAAAAT----------------------------------------------TTCAATTTAAGTTTTTAATTTAAATAAATAAAGATTAGGCTCTGTAATGGTATGGGGTTTGTGCCCCA---------------------------   738
aa9_A08     -----------------------------------------------------CCCCCCAAAA---------------------------------------------------------------------------------------   663
aa39_D02    --------------------------------------------------------CCCAAA----------------------------------------------------------------------------------------   662
aa41_D04    ATAACAC-----------------------------------------------TCCCCAATGGTATGGGGTTTGTGCCCA---------------------------------------------------------------------   697
aa15_B02    CAATGCACCCATGC----------------------------------------------------------------------------------------------------------------------------------------   674
aa52_E03    CA----------------------------------------------------------------------------------------------------------------------------------------------------   664
aa7_A06     ------------------------------------------------------------------------------------------------------------------------------------------------------   659
aa23_B10    CCCAAACA----------------------------------------------------------------------------------------------------------------------------------------------   668
aa50_E01    CCAAGGAGT---------------------------------------------------------------------------------------------------------------------------------------------   672
            ......1360......1370......1380......1390......1400......1410......1420......1430......1440......1450......1460......1470......1480......1490......1500
```

```
aa12_A11    ------------------------------------------------------------------------------------------------------------------------------------------------------   661
aa21_B08    ------------------------------------------------------------------------------------------------------------------------------------------------------   661
aa24_B11    ------------------------------------------------------------------------------------------------------------------------------------------------------   673
aa11_A10    ------------------------------------------------------------------------------------------------------------------------------------------------------   661
aa25_B12    ------------------------------------------------------------------------------------------------------------------------------------------------------   660
aa13_A12    ------------------------------------------------------------------------------------------------------------------------------------------------------   659
aa38_D01    ------------------------------------------------------------------------------------------------------------------------------------------------------   663
aa45_D08    ------------------------------------------------------------------------------------------------------------------------------------------------------   660
aa48_D11    ------------------------------------------------------------------------------------------------------------------------------------------------------   661
aa31_C06    ------------------------------------------------------------------------------------------------------------------------------------------------------   663
aa49_D12    ------------------------------------------------------------------------------------------------------------------------------------------------------   666
aa10_A09    ------------------------------------------------------------------------------------------------------------------------------------------------------   660
aa18_B05    ------------------------------------------------------------------------------------------------------------------------------------------------------   659
aa22_B09    ------------------------------------------------------------------------------------------------------------------------------------------------------   664
aa8_A07     ------------------------------------------------------------------------------------------------------------------------------------------------------   658
aa17_B04    ------------------------------------------------------------------------------------------------------------------------------------------------------   665
aa56_E07    ------------------------------------------------------------------------------------------------------------------------------------------------------   660
aa54_E05    ------------------------------------------------------------------------------------------------------------------------------------------------------   661
aa16_B03    ------------------------------------------------------------------------------------------------------------------------------------------------------   660
aa53_E04    ------------------------------------------------------------------------------------------------------------------------------------------------------   657
aa32_C07    ------------------------------------------------------------------------------------------------------------------------------------------------------   660
aa26_C01    ------------------------------------------------------------------------------------------------------------------------------------------------------   660
aa34_C09    ------------------------------------------------------------------------------------------------------------------------------------------------------   657
aa6_A05     ------------------------------------------------------------------------------------------------------------------------------------------------------   658
ncaa2_A02   ------------------------------------------------------------------------------------------------------------------------------------------------------   355
aa55_E06    ------------------------------------------------------------------------------------------------------------------------------------------------------   668
aa42_D05    ------------------------------------------------------------------------------------------------------------------------------------------------------   662
NC_AA_4_D10 ------------------------------------------------------------------------------------------------------------------------------------------------------   353
aa37_C12    ------------------------------------------------------------------------------------------------------------------------------------------------------   667
aa19_B06    ------------------------------------------------------------------------------------------------------------------------------------------------------   660
aa33_C08    ------------------------------------------------------------------------------------------------------------------------------------------------------   662
NC_AA_1_A10 ------------------------------------------------------------------------------------------------------------------------------------------------------   354
aa28_C03    ------------------------------------------------------------------------------------------------------------------------------------------------------   659
aa29_C04    ------------------------------------------------------------------------------------------------------------------------------------------------------   660
aa30_C05    ------------------------------------------------------------------------------------------------------------------------------------------------------   655
aa43_D06    ------------------------------------------------------------------------------------------------------------------------------------------------------   679
NC_AA_5_E10 ------------------------------------------------------------------------------------------------------------------------------------------------------   354
aa57_E08    ------------------------------------------------------------------------------------------------------------------------------------------------------   686
NC_AA_2_B10 ------------------------------------------------------------------------------------------------------------------------------------------------------   366
aa40_D03    ------------------------------------------------------------------------------------------------------------------------------------------------------   664
aa44_D07    ------------------------------------------------------------------------------------------------------------------------------------------------------   671
aa47_D10    ------------------------------------------------------------------------------------------------------------------------------------------------------   664
aa20_B07    ------------------------------------------------------------------------------------------------------------------------------------------------------   669
aa46_D09    ------------------------------------------------------------------------------------------------------------------------------------------------------   668
aa14_B01    ------------------------------------------------------------------------------------------------------------------------------------------------------   664
aa35_C10    ------------------------------------------------------------------------------------------------------------------------------------------------------   665
aa36_C11    ------------------------------------------------------------------------------------------------------------------------------------------------------   668
aa58_E09    ------------------------------------------------------------------------------------------------------------------------------------------------------   663
aa51_E02    ------------------------------------------------------------------------------------------------------------------------------------------------------   738
aa9_A08     ------------------------------------------------------------------------------------------------------------------------------------------------------   663
aa39_D02    ------------------------------------------------------------------------------------------------------------------------------------------------------   662
aa41_D04    ------------------------------------------------------------------------------------------------------------------------------------------------------   697
aa15_B02    ------------------------------------------------------------------------------------------------------------------------------------------------------   674
aa52_E03    ------------------------------------------------------------------------------------------------------------------------------------------------------   664
aa7_A06     ------------------------------------------------------------------------------------------------------------------------------------------------------   659
aa23_B10    ------------------------------------------------------------------------------------------------------------------------------------------------------   668
aa50_E01    ------------------------------------------------------------------------------------------------------------------------------------------------------   672
            ......1510......1520......1530......1540......1550......1560......1570......1580......1590......1600......1610......1620......1630......1640......1650
```

```
aa12_A11     ------------------------------------------------------------------------------------------------------------------------------------------------------   661
aa21_B08     ------------------------------------------------------------------------------------------------------------------------------------------------------   661
aa24_B11     ------------------------------------------------------------------------------------------------------------------------------------------------------   673
aa11_A10     ------------------------------------------------------------------------------------------------------------------------------------------------------   661
aa25_B12     ------------------------------------------------------------------------------------------------------------------------------------------------------   660
aa13_A12     ------------------------------------------------------------------------------------------------------------------------------------------------------   659
aa38_D01     ------------------------------------------------------------------------------------------------------------------------------------------------------   663
aa45_D08     ------------------------------------------------------------------------------------------------------------------------------------------------------   660
aa48_D11     ------------------------------------------------------------------------------------------------------------------------------------------------------   661
aa31_C06     ------------------------------------------------------------------------------------------------------------------------------------------------------   663
aa49_D12     ------------------------------------------------------------------------------------------------------------------------------------------------------   666
aa10_A09     ------------------------------------------------------------------------------------------------------------------------------------------------------   660
aa18_B05     ------------------------------------------------------------------------------------------------------------------------------------------------------   659
aa22_B09     ------------------------------------------------------------------------------------------------------------------------------------------------------   664
aa8_A07      ------------------------------------------------------------------------------------------------------------------------------------------------------   658
aa17_B04     ------------------------------------------------------------------------------------------------------------------------------------------------------   665
aa56_E07     ------------------------------------------------------------------------------------------------------------------------------------------------------   660
aa54_E05     ------------------------------------------------------------------------------------------------------------------------------------------------------   661
aa16_B03     ------------------------------------------------------------------------------------------------------------------------------------------------------   660
aa53_E04     ------------------------------------------------------------------------------------------------------------------------------------------------------   657
aa32_C07     ------------------------------------------------------------------------------------------------------------------------------------------------------   660
aa26_C01     ------------------------------------------------------------------------------------------------------------------------------------------------------   660
aa34_C09     ------------------------------------------------------------------------------------------------------------------------------------------------------   657
aa6_A05      ------------------------------------------------------------------------------------------------------------------------------------------------------   658
ncaa2_A02    ------------------------------------------------------------------------------------------------------------------------------------------------------   355
aa55_E06     ------------------------------------------------------------------------------------------------------------------------------------------------------   668
aa42_D05     ------------------------------------------------------------------------------------------------------------------------------------------------------   662
NC_AA_4_D10  ------------------------------------------------------------------------------------------------------------------------------------------------------   353
aa37_C12     ------------------------------------------------------------------------------------------------------------------------------------------------------   667
aa19_B06     ------------------------------------------------------------------------------------------------------------------------------------------------------   660
aa33_C08     ------------------------------------------------------------------------------------------------------------------------------------------------------   662
NC_AA_1_A10  ------------------------------------------------------------------------------------------------------------------------------------------------------   354
aa28_C03     ------------------------------------------------------------------------------------------------------------------------------------------------------   659
aa29_C04     ------------------------------------------------------------------------------------------------------------------------------------------------------   660
aa30_C05     ------------------------------------------------------------------------------------------------------------------------------------------------------   655
aa43_D06     ------------------------------------------------------------------------------------------------------------------------------------------------------   679
NC_AA_5_E10  ------------------------------------------------------------------------------------------------------------------------------------------------------   354
aa57_E08     ------------------------------------------------------------------------------------------------------------------------------------------------------   686
NC_AA_2_B10  ------------------------------------------------------------------------------------------------------------------------------------------------------   366
aa40_D03     ------------------------------------------------------------------------------------------------------------------------------------------------------   664
aa44_D07     ------------------------------------------------------------------------------------------------------------------------------------------------------   671
aa47_D10     ------------------------------------------------------------------------------------------------------------------------------------------------------   664
aa20_B07     ------------------------------------------------------------------------------------------------------------------------------------------------------   669
aa46_D09     ------------------------------------------------------------------------------------------------------------------------------------------------------   668
aa14_B01     ------------------------------------------------------------------------------------------------------------------------------------------------------   664
aa35_C10     ------------------------------------------------------------------------------------------------------------------------------------------------------   665
aa36_C11     ------------------------------------------------------------------------------------------------------------------------------------------------------   668
aa58_E09     ------------------------------------------------------------------------------------------------------------------------------------------------------   663
aa51_E02     ------------------------------------------------------------------------------------------------------------------------------------------------------   738
aa9_A08      ------------------------------------------------------------------------------------------------------------------------------------------------------   663
aa39_D02     ------------------------------------------------------------------------------------------------------------------------------------------------------   662
aa41_D04     ------------------------------------------------------------------------------------------------------------------------------------------------------   697
aa15_B02     ------------------------------------------------------------------------------------------------------------------------------------------------------   674
aa52_E03     ------------------------------------------------------------------------------------------------------------------------------------------------------   664
aa7_A06      ------------------------------------------------------------------------------------------------------------------------------------------------------   659
aa23_B10     ------------------------------------------------------------------------------------------------------------------------------------------------------   668
aa50_E01     ------------------------------------------------------------------------------------------------------------------------------------------------------   672
             ......1660......1670......1680......1690......1700......1710......1720......1730......1740......1750......1760......1770......1780......1790......1800
```

```
aa12_A11      ------------------------------------------------------------------------------------------------------------------------------------------------------  661
aa21_B08      ------------------------------------------------------------------------------------------------------------------------------------------------------  661
aa24_B11      ------------------------------------------------------------------------------------------------------------------------------------------------------  673
aa11_A10      ------------------------------------------------------------------------------------------------------------------------------------------------------  661
aa25_B12      ------------------------------------------------------------------------------------------------------------------------------------------------------  660
aa13_A12      ------------------------------------------------------------------------------------------------------------------------------------------------------  659
aa38_D01      ------------------------------------------------------------------------------------------------------------------------------------------------------  663
aa45_D08      ------------------------------------------------------------------------------------------------------------------------------------------------------  660
aa48_D11      ------------------------------------------------------------------------------------------------------------------------------------------------------  661
aa31_C06      ------------------------------------------------------------------------------------------------------------------------------------------------------  663
aa49_D12      ------------------------------------------------------------------------------------------------------------------------------------------------------  666
aa10_A09      ------------------------------------------------------------------------------------------------------------------------------------------------------  660
aa18_B05      ------------------------------------------------------------------------------------------------------------------------------------------------------  659
aa22_B09      ------------------------------------------------------------------------------------------------------------------------------------------------------  664
aa8_A07       ------------------------------------------------------------------------------------------------------------------------------------------------------  658
aa17_B04      ------------------------------------------------------------------------------------------------------------------------------------------------------  665
aa56_E07      ------------------------------------------------------------------------------------------------------------------------------------------------------  660
aa54_E05      ------------------------------------------------------------------------------------------------------------------------------------------------------  661
aa16_B03      ------------------------------------------------------------------------------------------------------------------------------------------------------  660
aa53_E04      ------------------------------------------------------------------------------------------------------------------------------------------------------  657
aa32_C07      ------------------------------------------------------------------------------------------------------------------------------------------------------  660
aa26_C01      ------------------------------------------------------------------------------------------------------------------------------------------------------  660
aa34_C09      ------------------------------------------------------------------------------------------------------------------------------------------------------  657
aa6_A05       ------------------------------------------------------------------------------------------------------------------------------------------------------  658
ncaa2_A02     ------------------------------------------------------------------------------------------------------------------------------------------------------  355
aa55_E06      ------------------------------------------------------------------------------------------------------------------------------------------------------  668
aa42_D05      ------------------------------------------------------------------------------------------------------------------------------------------------------  662
NC_AA_4_D10   ------------------------------------------------------------------------------------------------------------------------------------------------------  353
aa37_C12      ------------------------------------------------------------------------------------------------------------------------------------------------------  667
aa19_B06      ------------------------------------------------------------------------------------------------------------------------------------------------------  660
aa33_C08      ------------------------------------------------------------------------------------------------------------------------------------------------------  662
NC_AA_1_A10   ------------------------------------------------------------------------------------------------------------------------------------------------------  354
aa28_C03      ------------------------------------------------------------------------------------------------------------------------------------------------------  659
aa29_C04      ------------------------------------------------------------------------------------------------------------------------------------------------------  660
aa30_C05      ------------------------------------------------------------------------------------------------------------------------------------------------------  655
aa43_D06      ------------------------------------------------------------------------------------------------------------------------------------------------------  679
NC_AA_5_E10   ------------------------------------------------------------------------------------------------------------------------------------------------------  354
aa57_E08      ------------------------------------------------------------------------------------------------------------------------------------------------------  686
NC_AA_2_B10   ------------------------------------------------------------------------------------------------------------------------------------------------------  366
aa40_D03      ------------------------------------------------------------------------------------------------------------------------------------------------------  664
aa44_D07      ------------------------------------------------------------------------------------------------------------------------------------------------------  671
aa47_D10      ------------------------------------------------------------------------------------------------------------------------------------------------------  664
aa20_B07      ------------------------------------------------------------------------------------------------------------------------------------------------------  669
aa46_D09      ------------------------------------------------------------------------------------------------------------------------------------------------------  668
aa14_B01      ------------------------------------------------------------------------------------------------------------------------------------------------------  664
aa35_C10      ------------------------------------------------------------------------------------------------------------------------------------------------------  665
aa36_C11      ------------------------------------------------------------------------------------------------------------------------------------------------------  668
aa58_E09      ------------------------------------------------------------------------------------------------------------------------------------------------------  663
aa51_E02      ------------------------------------------------------------------------------------------------------------------------------------------------------  738
aa9_A08       ------------------------------------------------------------------------------------------------------------------------------------------------------  663
aa39_D02      ------------------------------------------------------------------------------------------------------------------------------------------------------  662
aa41_D04      ------------------------------------------------------------------------------------------------------------------------------------------------------  697
aa15_B02      ------------------------------------------------------------------------------------------------------------------------------------------------------  674
aa52_E03      ------------------------------------------------------------------------------------------------------------------------------------------------------  664
aa7_A06       ------------------------------------------------------------------------------------------------------------------------------------------------------  659
aa23_B10      ------------------------------------------------------------------------------------------------------------------------------------------------------  668
aa50_E01      ------------------------------------------------------------------------------------------------------------------------------------------------------  672
              ......1810......1820......1830......1840......1850......1860......1870......1880......1890......1900......1910......1920......1930......1940......1950
```

```
aa12_A11     ------------------------------------------------------------------------------------------------------------------------------------------------------   661
aa21_B08     ------------------------------------------------------------------------------------------------------------------------------------------------------   661
aa24_B11     ------------------------------------------------------------------------------------------------------------------------------------------------------   673
aa11_A10     ------------------------------------------------------------------------------------------------------------------------------------------------------   661
aa25_B12     ------------------------------------------------------------------------------------------------------------------------------------------------------   660
aa13_A12     ------------------------------------------------------------------------------------------------------------------------------------------------------   659
aa38_D01     ------------------------------------------------------------------------------------------------------------------------------------------------------   663
aa45_D08     ------------------------------------------------------------------------------------------------------------------------------------------------------   660
aa48_D11     ------------------------------------------------------------------------------------------------------------------------------------------------------   661
aa31_C06     ------------------------------------------------------------------------------------------------------------------------------------------------------   663
aa49_D12     ------------------------------------------------------------------------------------------------------------------------------------------------------   666
aa10_A09     ------------------------------------------------------------------------------------------------------------------------------------------------------   660
aa18_B05     ------------------------------------------------------------------------------------------------------------------------------------------------------   659
aa22_B09     ------------------------------------------------------------------------------------------------------------------------------------------------------   664
aa8_A07      ------------------------------------------------------------------------------------------------------------------------------------------------------   658
aa17_B04     ------------------------------------------------------------------------------------------------------------------------------------------------------   665
aa56_E07     ------------------------------------------------------------------------------------------------------------------------------------------------------   660
aa54_E05     ------------------------------------------------------------------------------------------------------------------------------------------------------   661
aa16_B03     ------------------------------------------------------------------------------------------------------------------------------------------------------   660
aa53_E04     ------------------------------------------------------------------------------------------------------------------------------------------------------   657
aa32_C07     ------------------------------------------------------------------------------------------------------------------------------------------------------   660
aa26_C01     ------------------------------------------------------------------------------------------------------------------------------------------------------   660
aa34_C09     ------------------------------------------------------------------------------------------------------------------------------------------------------   657
aa6_A05      ------------------------------------------------------------------------------------------------------------------------------------------------------   658
ncaa2_A02    ------------------------------------------------------------------------------------------------------------------------------------------------------   355
aa55_E06     ------------------------------------------------------------------------------------------------------------------------------------------------------   668
aa42_D05     ------------------------------------------------------------------------------------------------------------------------------------------------------   662
NC_AA_4_D10  ------------------------------------------------------------------------------------------------------------------------------------------------------   353
aa37_C12     ------------------------------------------------------------------------------------------------------------------------------------------------------   667
aa19_B06     ------------------------------------------------------------------------------------------------------------------------------------------------------   660
aa33_C08     ------------------------------------------------------------------------------------------------------------------------------------------------------   662
NC_AA_1_A10  ------------------------------------------------------------------------------------------------------------------------------------------------------   354
aa28_C03     ------------------------------------------------------------------------------------------------------------------------------------------------------   659
aa29_C04     ------------------------------------------------------------------------------------------------------------------------------------------------------   660
aa30_C05     ------------------------------------------------------------------------------------------------------------------------------------------------------   655
aa43_D06     ------------------------------------------------------------------------------------------------------------------------------------------------------   679
NC_AA_5_E10  ------------------------------------------------------------------------------------------------------------------------------------------------------   354
aa57_E08     ------------------------------------------------------------------------------------------------------------------------------------------------------   686
NC_AA_2_B10  ------------------------------------------------------------------------------------------------------------------------------------------------------   366
aa40_D03     ------------------------------------------------------------------------------------------------------------------------------------------------------   664
aa44_D07     ------------------------------------------------------------------------------------------------------------------------------------------------------   671
aa47_D10     ------------------------------------------------------------------------------------------------------------------------------------------------------   664
aa20_B07     ------------------------------------------------------------------------------------------------------------------------------------------------------   669
aa46_D09     ------------------------------------------------------------------------------------------------------------------------------------------------------   668
aa14_B01     ------------------------------------------------------------------------------------------------------------------------------------------------------   664
aa35_C10     ------------------------------------------------------------------------------------------------------------------------------------------------------   665
aa36_C11     ------------------------------------------------------------------------------------------------------------------------------------------------------   668
aa58_E09     ------------------------------------------------------------------------------------------------------------------------------------------------------   663
aa51_E02     ------------------------------------------------------------------------------------------------------------------------------------------------------   738
aa9_A08      ------------------------------------------------------------------------------------------------------------------------------------------------------   663
aa39_D02     ------------------------------------------------------------------------------------------------------------------------------------------------------   662
aa41_D04     ------------------------------------------------------------------------------------------------------------------------------------------------------   697
aa15_B02     ------------------------------------------------------------------------------------------------------------------------------------------------------   674
aa52_E03     ------------------------------------------------------------------------------------------------------------------------------------------------------   664
aa7_A06      ------------------------------------------------------------------------------------------------------------------------------------------------------   659
aa23_B10     ------------------------------------------------------------------------------------------------------------------------------------------------------   668
aa50_E01     ------------------------------------------------------------------------------------------------------------------------------------------------------   672
             ......1960......1970......1980......1990......2000......2010......2020......2030......2040......2050......2060......2070......2080......2090......2100
```

```
aa12_A11     ----------------------   661
aa21_B08     ----------------------   661
aa24_B11     ----------------------   673
aa11_A10     ----------------------   661
aa25_B12     ----------------------   660
aa13_A12     ----------------------   659
aa38_D01     ----------------------   663
aa45_D08     ----------------------   660
aa48_D11     ----------------------   661
aa31_C06     ----------------------   663
aa49_D12     ----------------------   666
aa10_A09     ----------------------   660
aa18_B05     ----------------------   659
aa22_B09     ----------------------   664
aa8_A07      ----------------------   658
aa17_B04     ----------------------   665
aa56_E07     ----------------------   660
aa54_E05     ----------------------   661
aa16_B03     ----------------------   660
aa53_E04     ----------------------   657
aa32_C07     ----------------------   660
aa26_C01     ----------------------   660
aa34_C09     ----------------------   657
aa6_A05      ----------------------   658
ncaa2_A02    ----------------------   355
aa55_E06     ----------------------   668
aa42_D05     ----------------------   662
NC_AA_4_D10  ----------------------   353
aa37_C12     ----------------------   667
aa19_B06     ----------------------   660
aa33_C08     ----------------------   662
NC_AA_1_A10  ----------------------   354
aa28_C03     ----------------------   659
aa29_C04     ----------------------   660
aa30_C05     ----------------------   655
aa43_D06     ----------------------   679
NC_AA_5_E10  ----------------------   354
aa57_E08     ----------------------   686
NC_AA_2_B10  ----------------------   366
aa40_D03     ----------------------   664
aa44_D07     ----------------------   671
aa47_D10     ----------------------   664
aa20_B07     ----------------------   669
aa46_D09     ----------------------   668
aa14_B01     ----------------------   664
aa35_C10     ----------------------   665
aa36_C11     ----------------------   668
aa58_E09     ----------------------   663
aa51_E02     ----------------------   738
aa9_A08      ----------------------   663
aa39_D02     ----------------------   662
aa41_D04     ----------------------   697
aa15_B02     ----------------------   674
aa52_E03     ----------------------   664
aa7_A06      ----------------------   659
aa23_B10     ----------------------   668
aa50_E01     ----------------------   672
             ......2110......2120..
```
